# Role of Exosome in Solid Cancer Progression and Its Potential Therapeutics in Cancer Treatment

**DOI:** 10.1002/cam4.70941

**Published:** 2025-05-09

**Authors:** Nada Aakel, Rawdhah Mohammed, Assela Fathima, Rabia Kerzabi, Atiyeh Abdallah, Wisam Nabeel Ibrahim

**Affiliations:** ^1^ Department of Biomedical Science College of Health Sciences, QU Health, Qatar University Doha Qatar

**Keywords:** cancer biology, cancer progression, exosomes, extracellular vesicles, molecular signaling, solid tumors, therapeutic targets, tumor microenvironment

## Abstract

**Background:**

Exosomes are extracellular vesicles ranging from 40 to 100 nm in diameter that mediate intercellular communication by transferring proteins, lipids, nucleic acids, and other metabolites. In the context of cancer, exosomes influence the tumor microenvironment by carrying regulatory RNAs such as miRNA, circRNA, and lncRNA. They originate from various cells, including adipocytes, fibroblasts, and hepatocellular carcinoma (HCC) cells, and can either promote or inhibit cancer progression through pathways like MAPK and PI3K‐Akt.

**Aim:**

This review aims to explore the role of exosomes in the progression of solid cancers, emphasizing their self‐induced activation mechanisms and how they modulate tumor behavior.

**Methodology:**

A comprehensive review of recent literature was conducted, focusing on studies that investigated the biological functions of exosomes in solid tumor progression, including their molecular cargo, cellular origin, and involvement in signaling pathways.

**Results:**

Findings from multiple studies indicate that cancer‐derived exosomes contribute to tumor proliferation, metastasis, and therapy resistance by enhancing communication within the tumor microenvironment. These vesicles activate oncogenic pathways and can serve as biomarkers or therapeutic targets due to their role in disease modulation.

**Conclusion:**

Exosomes play a pivotal role in solid cancer progression and offer significant potential in advancing our understanding of tumor biology. Their capacity to influence key signaling pathways and facilitate intercellular communication makes them promising candidates for novel diagnostic and therapeutic strategies.

## Graphical abstract

Exosomes are small extracellular vesicles that facilitate cellular communication and play a significant role in cancer progression by transferring molecules like miRNA and lncRNA within the tumor microenvironment. This review explores how exosomes derived from various cells influence solid cancer growth by activating pathways critical to tumor proliferation and metastasis. Understanding these interactions may reveal new therapeutic approaches for treating solid tumors.

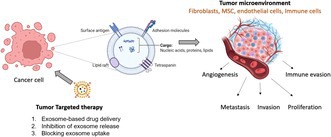

## 1 Introduction

Cancer, once relatively uncommon, has seen a significant rise in incidence over the past two decades, driven by lifestyle changes, evolving habits, and increased life expectancy [1]. It is a disease characterized by gene mutations that disrupt normal cellular processes of replication, differentiation, and death, leading to the initiation and progression of tumors [2]. As tumors grow, they recruit normal cells from the surrounding environment, causing structural and cellular changes that impact tumor biology and response to treatment [3]. Standard treatments, such as surgery, radiation, and chemotherapy, can damage healthy cells and cause toxic side effects [4]. Thus, developing more effective and precise cancer therapies is crucial for enhancing patient outcomes.

The tumor microenvironment (TME) is a complex network comprising malignant cells and various supportive components, including fibroblasts, adipocytes, endothelial cells, immune cells, and the extracellular matrix (ECM) [5]. Cells within the TME interact through direct contact and the release of signaling molecules like cytokines, chemokines, and extracellular vesicles (EVs) such as exosomes [6]. Exosomes, once released, are internalized by recipient cells through endocytosis or receptor‐mediated interactions, facilitating intercellular communication [7, 8]. This signaling can drive immune evasion, alter stromal cell behavior, and remodel the ECM, promoting tumor progression [9, 10, 11].

Exosomes are EVs released via exocytosis, resulting in a lipid bilayer orientation similar to the plasma membrane. Markers of exosome biogenesis include proteins such as Rab GTPases and ESCRT proteins, with commonly recognized surface markers being CD9, CD81, CD63, flotillin, TSG101, ceramide, and Alix [12]. Exosomes, classified based on size and origin, range from 40 to 100 nm in diameter and originate from endosomes. Larger EVs like macrovesicles and apoptotic bodies are derived from the plasma membrane [13]. Exosomes can also be categorized based on their cellular origin, such as adipocyte‐derived, fibroblast‐derived, or stem cell‐derived exosomes, each playing roles as either cancer promoters or inhibitors [14, 15, 16, 17].

Exosomes have emerged as a significant focus of research in cancer biology due to their dual nature. They are implicated in tumor progression through the transfer of miRNA, circRNA, and lncRNA, which activate pathways like MAPK, RAS, PI3K‐Akt, and Wnt/β‐catenin, promoting cancer proliferation and metastasis [15, 18]. Exosomes also contribute to tumor immunosuppression by altering immune responses, such as transforming T‐cells into Treg cells and inducing NK cell exhaustion [19, 20, 21]. Conversely, some exosomes inhibit cancer growth, as seen with umbilical cord mesenchymal stem cell (MSC)‐derived exosomes that suppress hepatocellular carcinoma (HCC) proliferation, induce apoptosis, and reduce angiogenesis [14]. Other exosomes, such as those containing circ_0051443, promote apoptosis and cell cycle arrest in cancer cells, highlighting their potential therapeutic role [22].

This review aims to update our present understanding of the biogenesis and functions of tumor‐derived exosomes, emphasizing their critical contribution to TME signaling and their influence on solid cancer progression, focusing on brain, liver, and breast cancers. These cancers were selected for their obvious clinical and biologic relevance: Brain tumors, i.e., glioblastoma (GBM), represent a particular therapeutic challenge due to the blood–brain barrier (BBB) and immunosuppressive TME, but also offer the opportunity for exosome‐mediated drug delivery, as discussed under therapeutic approaches [23, 24]. Liver cancers, including HCC, are intimately associated with chronic inflammation and fibrosis, where exosomes facilitate immune evasion and stromal remodeling to sustain a pro‐tumorigenic niche [25, 26]. Breast cancer, with its astounding heterogeneity, is an exemplary model of exosome‐mediated communication between tumor cells and stromal components such as adipocytes, macrophages, and fibroblasts, which fuel metastasis and therapy resistance [27, 28]. Collectively, these malignancies highlight diverse microenvironments and exosome‐mediated processes that are primed for biomarker development and therapeutic innovation. By examining these environments, we interpret the double‐edged nature of exosomes as both architects of cancer and therapeutics, and their potential to refine targeted therapy strategies across solid tumors.

### 1.1 Biogenesis and Signaling Pathways of Tumor‐Derived Exosomes

Tumor‐derived exosomes (TDEs) have gained significant attention in cancer research due to their immunoregulatory roles and involvement in tumor pathophysiology. The biogenesis of exosomes involves four main stages: cargo sorting, endocytosis, multivesicular body (MVB) formation, and exosome release. This process begins with the recruitment of various molecules—proteins, nucleic acids, and lipids—into early endosomes. Cargo sorting is regulated by distinct mechanisms; for example, proteins often require monoubiquitination, while miRNAs are guided into exosomes by binding to heterogeneous nuclear ribonucleoprotein A2B1 (hnRNPA2B1) [29, 30, 31, 32].

**FIGURE 1:**
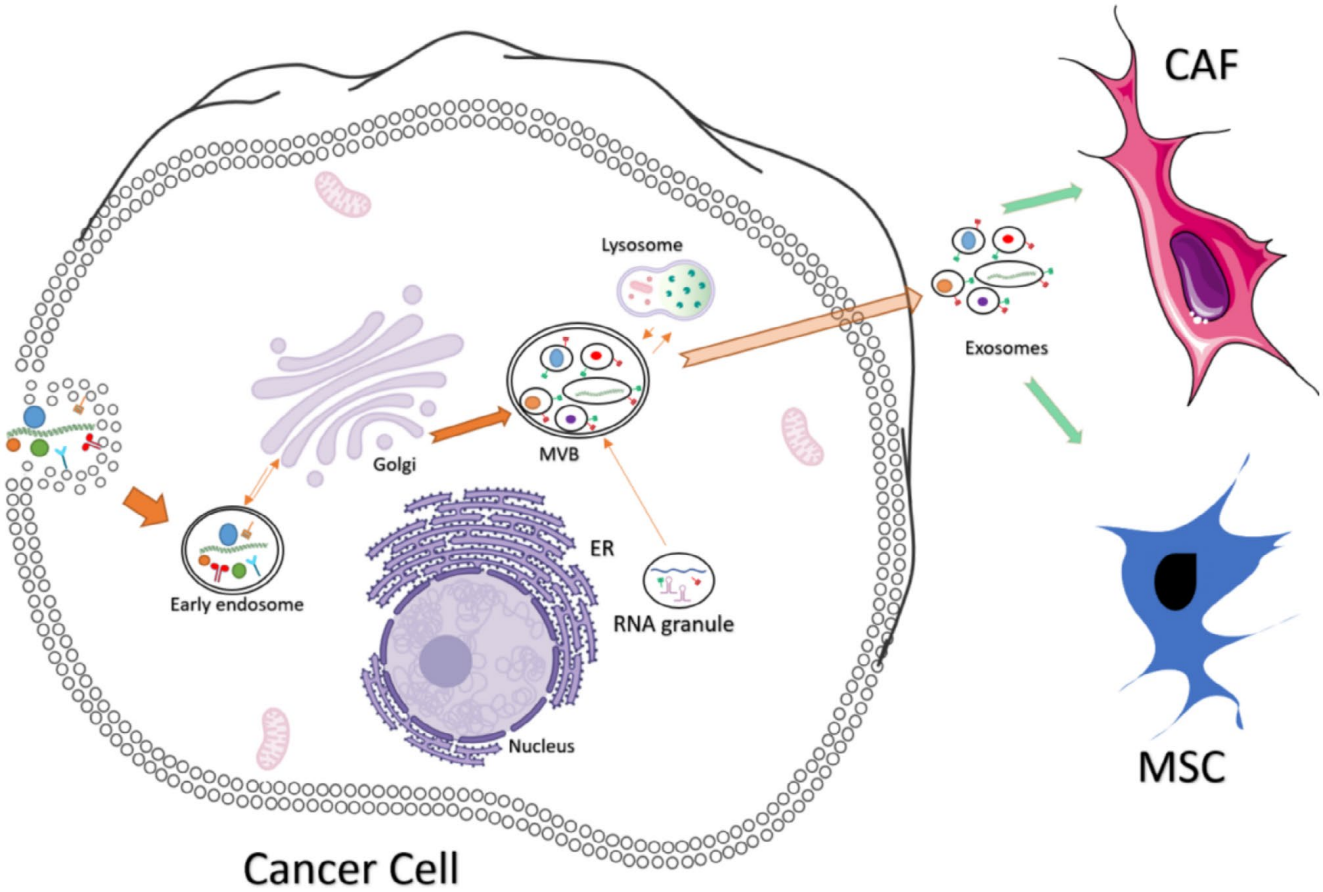
Exosomes biogenesis. Exosomes progress through phases of endocytosis and multivesicular body formation, where they can fuse with lysosomes or the plasma membrane for release. Cargo, including DNA, microRNA, and proteins, is packaged into exosomes, which then deliver their contents to stromal cells in the tumor microenvironment like cancer associated fibroblasts (CAF) or Mesenchymal stem cells (MSC).

Early endosomes form through plasma membrane invagination and undergo maturation into late endosomes, which involves changes in membrane composition, such as the conversion of sphingomyelin to ceramides and the replacement of Rab5 with Rab11, facilitating endosomal trafficking [33, 34, 35, 36]. During this maturation, inward budding of the endosomal membrane creates dynamic MVBs. These MVBs can either fuse with lysosomes, leading to cargo degradation, or with the plasma membrane, releasing their intraluminal vesicles (ILVs) as exosomes into the extracellular space [37, 38, 39, 40] as shown in Figure 1.

Exosome synthesis and release involve complex pathways, including Endosomal Sorting Complexes Required for Transport (ESCRT)‐dependent and ESCRT‐independent mechanisms. Released exosomes deliver their cargo—such as DNA, microRNA, and proteins—to recipient cells through mechanisms like direct fusion with the plasma membrane, surface protein binding, or endocytosis [41]. By utilizing these mechanisms, TDEs significantly contribute to intercellular communication, supporting tumor growth, proliferation, angiogenesis, and metastasis. They deliver immunosuppressive molecules to stromal and immune cells, dampening immune responses against cancer [42].

Exosomes derived from tumors express immunosuppressive proteins such as programmed death‐ligand 1 (PD‐L1), which inhibits T‐cell activation [43, 44]. TDEs also mediate immune suppression through the FAS/FASL pathway, promoting T‐cell apoptosis and signaling via exosomal TGF‐β1 [45]. The ESCRT‐associated protein ALIX regulates PD‐1 expression on cell membranes; its downregulation increases PD‐1 expression in breast cancer cells, enhancing immune evasion [46]. Cancer‐associated fibroblast (CAF)‐derived exosomes overexpress miR‐92, which upregulates PD‐L1 in breast cancer cells, linking reduced cell division and increased T‐cell apoptosis [47]. TDE signaling further suppresses natural killer (NK) cells by inhibiting interleukin‐2 and transporting heat shock protein‐72 to regulate myeloid‐derived suppressor cells (MDSCs) via the Signal transducer and activator of transcription 3 (STAT3) pathway. Additionally, TDEs transfer epidermal growth factor receptor (EGFR) to adjacent macrophages, contributing to immune suppression [46]. Exosomes from MSCs influence tumor‐associated macrophages (TAMs), converting them into immunosuppressive type‐2 macrophages that enhance arginase‐1 activity and interleukin‐10 secretion, promoting malignancy and immune suppression at the tumor site [48]. Exosomes deriving from MSCs have a large amount of semaphorins, TGF‐β and C1Q which incentivize the expression of PD‐L1 in macrophages [49]. The epithelial–mesenchymal transition (EMT) also stimulates exosome biogenesis and release in tumor cells. CAFs secrete TGF‐β1 through the TGF‐β1‐SMAD pathway, facilitating EMT and supporting exosome‐mediated signaling [50, 51, 52]. Exosomes from these fibroblasts can activate the Wnt pathway in breast cancer cells, promoting metastasis [53].

Exosome‐mediated delivery of miRNA‐rich cargo activates key pathways such as Ras, PI3K‐Akt, and MAPK, driving cancer proliferation [15]. For example, exosomes containing miR‐374a‐5p, miR‐200b‐3p, and miR‐21‐5p activate the Wnt/β‐catenin and PI3K/Akt pathways in HCC, enhancing tumor aggressiveness [15]. miR‐17‐5p, another exosomal miRNA, promotes proliferation by inhibiting MAPK9 and suppressing the G1/S cell cycle checkpoint [54]. Downregulation of critical mRNAs, like GADD45A, further destabilizes genomic integrity, contributing to aggressive cancer phenotypes [15]. Furthermore, genes associated with cell proliferation and collagen production were found to be upregulated, whereas those related to immune functions, such as CLEC1B and CLEC4G, were downregulated [16].

### 1.2 Tumor Microenvironment

**FIGURE 2:**
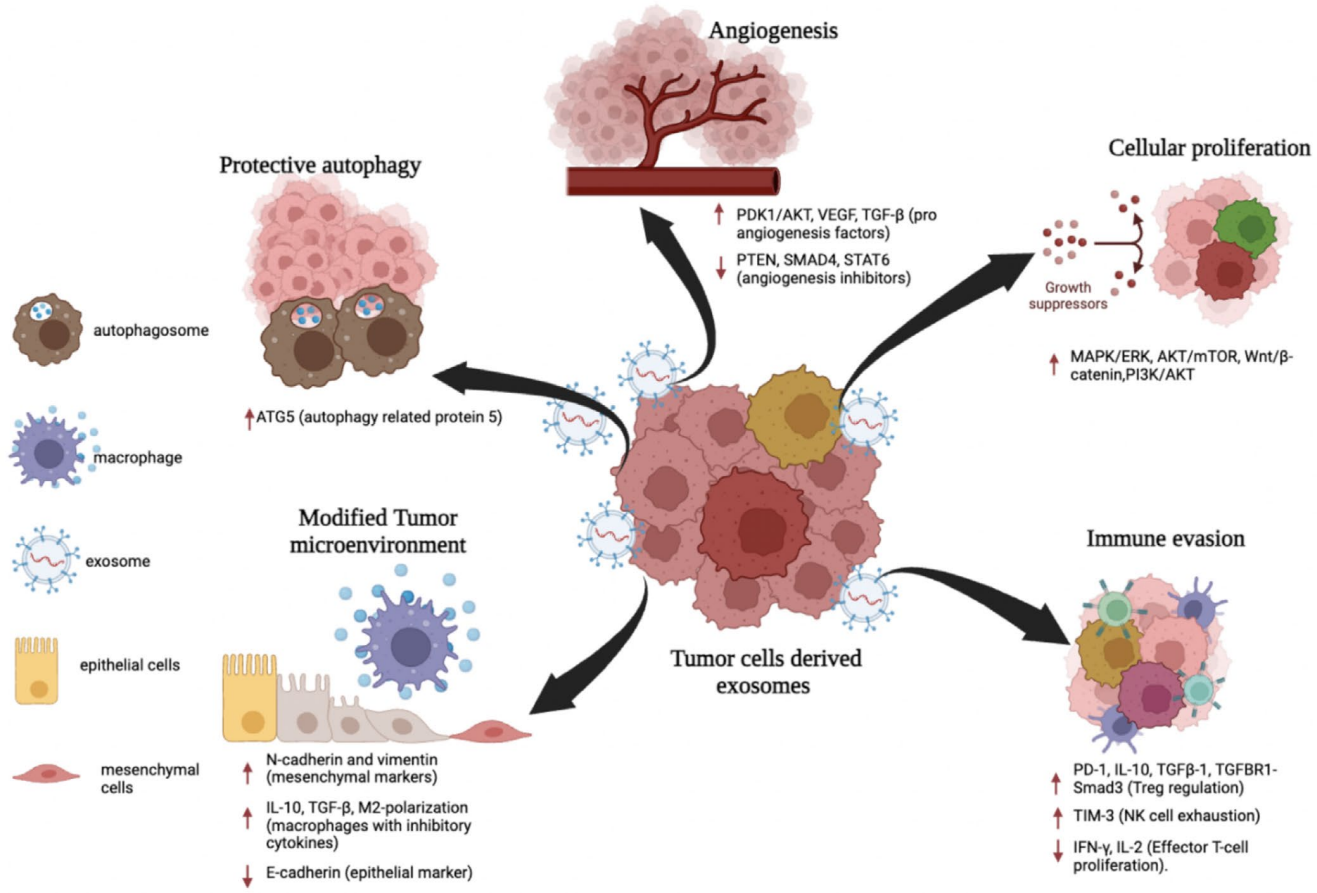
Role of exosomes in cancer progression. Exosomes containing cargo help in cancer metastasis through cellular proliferation, immune evasion, angiogenesis, protective autophagy and modifying the tumor microenvironment. Created with BioRender.com.

The TME is a dynamic and intricate ecosystem that evolves continuously to support cancer development. It consists of diverse cellular and non‐cellular components that play pivotal roles in all stages of carcinogenesis. The TME typically comprises immune cells, stromal cells, blood vessels, and the ECM, though its composition varies by tumor type [55, 56]. The interaction between cancer cells and the surrounding environment fosters tumor survival, local invasion, and metastatic spread from the early stages of tumor growth, illustrated in Figure 2 [55].

#### 1.2.1 Immune Cells in the TME

Immune cells within the TME can either fight against the tumor or facilitate its growth, contributing to a highly dynamic immune landscape. Tumors can be classified into three types based on immune infiltration: immune‐infiltrated, immune‐excluded (where immune cells are at the tumor's periphery but have not penetrated it), and immune‐silent (with a complete lack of immune cell infiltration) [55, 57]. In immune‐infiltrated tumors, cytotoxic T‐cells move via a multistep process from the bloodstream to the tumor, signifying an active immune response [58]. However, tumor‐associated immune cells, such as TAMs, often support tumor progression by promoting angiogenesis, suppressing T‐cell function, and aiding metastasis [55, 59].

#### 1.2.2 Stromal Cells and the ECM

Stromal cells, such as CAFs, adipocytes, and endothelial cells, play critical roles in tumor growth, angiogenesis, and metastasis. CAFs are particularly influential in restricting immune cell access to the tumor and secreting factors that support cancer cell survival [60]. Adipocytes in adipocyte‐rich environments, such as breast cancer, release metabolic substrates, and cytokines that promote cancer cell proliferation and migration [61, 62].

In liver cancer, hepatic stellate cells (HSCs) transform into myofibroblasts in response to tissue injury, contributing to the fibrotic and inflammatory environment that supports HCC development [63, 64]. Endothelial cells (ECs) in the vascular endothelium are essential for tumor angiogenesis, which restores oxygen and nutrient supply to growing tumors that have outgrown passive diffusion [65]. This angiogenic process is often driven by hypoxia and is critical to the survival of tumors larger than 1–2 mm^3^ [55, 65].

The ECM, a three‐dimensional network of macromolecules such as collagen, fibronectin, elastin, laminin, and proteoglycans, provides structural support to the tumor and plays a crucial role in facilitating metastasis [66]. ECM consists of collagen, fibronectin, elastin, laminin, glycoproteins, and proteoglycans [67]. In solid tumors, the ECM forms extensive deposits, making up as much as 60% of the tumor's mass [55, 68].

**FIGURE 3:**
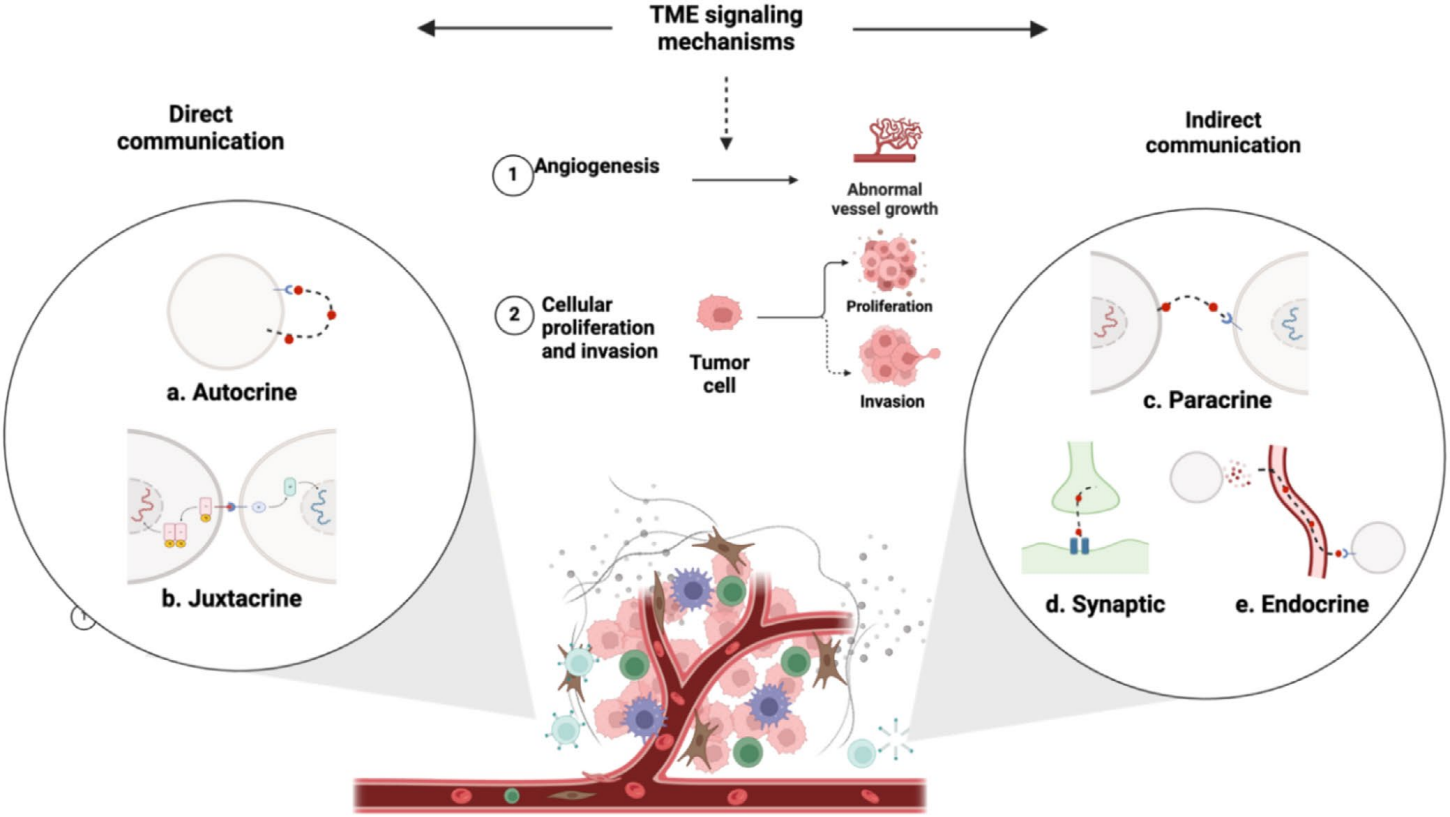
Illustrates the cell interactions that use various signaling mechanisms to form the tumor microenvironment (TME). These interactions include direct contact through such as (a) autocrine signaling, where a cell binds to its own secreted messenger, or (b) juxtacrine signaling, where membrane‐bound ligands on one cell interact with receptors on an adjacent cell. Alternatively, indirect communication occurs locally through (c) paracrine or (d) synaptic signaling, particularly in neural cancers such as brain cancer. Additionally, cells communicate over longer distances through (e) endocrine signaling mechanisms, including the transfer of biological information via exosomes or by releasing signaling molecules and mediators such as cytokines, chemokines, and growth factors. These interactions influence critical aspects of cancer, including immune response modulation, reprogramming of stromal cells, and remodeling of the extracellular matrix, ultimately leading to tumor progression through cellular proliferation, invasion, and angiogenesis. Created with BioRender.com.

In the TME, cells interact through various signaling mechanisms that promote carcinogenesis. These interactions can be direct, such as autocrine signaling where a cell binds to its own secreted messenger (Figure 3a) [69], or juxtacrine signaling where membrane‐bound ligands on one cell interact with receptors on an adjacent cell (Figure 3b) [70]. Alternatively, indirect communication occurs locally through paracrine –such as VEGF‐driven angiogenesis (Figure 3c) [71, 72], or synaptic signaling, particularly in neural cancers such as brain cancer [73] (Figure 3d). Additionally, cells communicate over longer distances through endocrine signaling mechanisms, including the transfer of biological information via exosomes [74, 75, 76], or by releasing signaling molecules and mediators such as cytokines, chemokines, and growth factors (Figure 3e) [75]. Consequently, these mechanisms modulate immune responses, reprogram stromal cells, and remodel the ECM, driving tumor proliferation, invasion, metastasis, and angiogenesis [9, 72, 77]. As tumors grow, hypoxia and metabolic waste accumulation create a stressful microenvironment that triggers angiogenesis to restore oxygen and nutrient levels [65]. Hypoxia‐induced factors, such as hypoxia‐inducible factor 1‐alpha (HIF‐1α), stimulate the secretion of pro‐angiogenic factors like VEGF, promoting the formation of new blood vessels. This newly formed vasculature is often abnormal, contributing to inefficient blood flow and exacerbating the hypoxic and acidic conditions within the TME, further driving tumor progression [65].

#### 1.2.3 Extracellular Vesicles in TME

EVs are crucial in promoting the process of cell–cell communication within the TME, initiating a cross talk between cancer cells and stromal cells [78]. EVs are classified into two major subgroups‐small vesicles budding directly from the endosomal compartments primarily called as exosomes and larger vesicles blebbing off the plasma membrane primarily termed as microvesicles [78]. EVs pathogenic process involves the transfer of the contained biomolecules either through fusing with the plasma membrane, endocytosis or ligand‐receptor interactions [79], promoting tumor growth, invasion, metastasis, angiogenesis, immunological modifications, EMT and inflammation, via transforming tumor cells, tumor‐associated cells (endothelial, fibroblast, macrophages, and leucocytes) or normal cells within the TME or at cells in distant locations developing pre‐metastatic niches [76, 80, 81]. The biomolecules utilized by the EVs could include proteins, lipids, DNAs, and non‐coding RNA [79]. For instance, EVs derived from breast cancer cells had an upregulated display of VEGF which stimulated the VEGFR‐signaling within the endothelial cells, with enhanced total VEGF secretion under hypoxic conditions promoting angiogenesis [81], or breast cancer cells derived EVs promoted epithelial to mesenchymal transition (EMT) by stimulating TGF‐β signaling due to the transfer of TGF‐beta II receptor, which is expressed on the EVs, promoting cancer stemness, metastasis and CD8+ T cells exhaustion by breast cancer cells [82]. However, most of the cell–cell communications are dominated by the non‐coding RNA (ncRNA) (miRNA, lnRNA, and circRNA) [83, 84]. For instance, EVs released by CAFs surrounding colorectal adenocarcinoma cells (COAD) promoted angiogenesis by delivering miR‐135‐5p, which negatively regulated FOXO1 expression, further inducing COAD cells to promote proliferation, migration and angiogenesis of endothelial cells causing angiogenesis [85]. The formation of the metastatic niche at a secondary location is primarily influenced by the EVs. For instance, the study conducted by Gener Lahav et al. investigated the potential of melanoma‐derived EVs to form secondary metastatic niches. They identified melanoma‐derived EVs increased proinflammatory signaling within astrocytes and lung fibroblast cells along with activated CAF characteristics, establishing hospitable metastatic niches [86]. Additionally, EVs derived from cells other than cancer cells promote tumorigenesis and modulate the TME [25]. The study conducted by Zhou et al. identified human umbilical mesenchymal stem cells (hU‐MSC) promoted proliferation, migration and EMT within breast cancer cells through the ERK pathway [25]. Furthermore, EVs are capable of destructing the blood‐brain barrier (BBB) promoting brain metastasis [87]. For instance, Tominaga et al. identified miR‐181c from the breast cancer cells promoted the destruction of BBB through the delocalization of actin through the downregulation of 3‐phosphoinositide‐dependent protein kinase‐1 (PDPK1), which resulted in the downregulation of phosphorylation of cofilin, leading to the modulation of actin dynamics, inducing brain metastasis. Therefore, EVs are derived from varied sources, such as cancer cells or from the surrounding cells, promoting cancer metastasis via modulating TME through cell‐to‐cell communication [87].

## 2 How EVs Control the Communication

Exosomes play a crucial role in cell–cell communication, traditionally mediated by mechanisms such as gap junctions, receptor/ligand interactions, and electrical or chemical signals. They help regulate cellular responses to the external environment. Numerous studies have confirmed the importance of exosomes in both physiological and pathological processes. TDEs are secreted in greater quantities by cancer cells than by normal cells, transferring tumor‐associated microRNAs and other signaling molecules to target cells through exosome fusion with cell membranes [88]. This exchange facilitates tumor growth, metastasis, and various other tumor‐promoting mechanisms, as illustrated in Figure 3 [89, 90].

For instance, in GBM multiforme, a highly aggressive brain tumor, exosomes have been shown to mediate specific communication between tumor cells and the surrounding non‐tumor cells, such as glial cells, neurons, and vascular cells. Longitudinal time‐lapse imaging revealed that glioma cells exchange EVs with different brain cell types to promote glioma growth. This communication is selective, with astrocytes or microglia/macrophages preferentially taking up debris from glioma cells. In a study where glioma‐derived EVs were injected into Ai14 mice, no significant EV uptake was observed in the brains of these animals, suggesting cell‐type specificity in EV‐mediated communication [91].

The proteins carried by TDEs significantly influence the TME. Among these proteins are cell adhesion molecules such as integrins, annexins, tetraspanins, and proteases. For example, Annexin A2 (ANXA2) plays a key role in invasion, metastasis, angiogenesis, and cell proliferation, while CD44, a receptor for hyaluronic acid, osteopontin, and matrix metalloproteinases, contributes to tumor growth, cell motility, and angiogenesis. CD44 is also regarded as a marker for cancer stem cells, further linking exosome‐mediated communication to malignancy [92].

TDEs play a crucial role not only in promoting tumor growth and progression but also in facilitating metastasis. For example, breast cancer‐derived exosomes have been shown to transport inducers of the EMT, a critical step in metastasis [93]. During EMT, epithelial cells lose their polarity and cell–cell adhesion properties, acquiring mesenchymal traits that allow them to migrate through the bloodstream, invade distant tissues, and form metastatic colonies [94]. This transition also leads to ECM remodeling and the development of anti‐apoptotic phenotypes and pre‐metastatic niches [95]. Moreover, TDEs facilitate organ‐specific metastasis. In gastric cancer, exosomes carrying EGFR have been implicated in promoting liver metastasis. These exosomes activate hepatocyte growth factor (HGF) by suppressing miR‐26a/b expression, thereby creating a liver‐like microenvironment. Elevated levels of HGF stimulate cancer cell proliferation through the activation of the c‐MET receptor, further promoting.

## 3 Exosomes and the Hallmarks of Cancer

EVs including exosomes, microvesicles, and apoptotic bodies, have emerged as key players in cancer research due to their involvement in the communication between cancer cells and the TME. Exosomes, in particular, are stable particles that can travel throughout the body, facilitating tumor growth and spread. Research has shown that both TDEs and stromal cell‐derived exosomes contribute to creating a supportive environment for cancer progression by modifying surrounding tissue [96]. EVs are essential for understanding the molecular mechanisms of cell communication within the TME, as they establish and maintain several cancer hallmarks, including promoting cell proliferation, resisting cell death, evading immune responses, reprogramming energy metabolism, and facilitating angiogenesis [97, 98, 99].

### 3.1 Promoting Cell Proliferation and Resisting Cell Death

EVs play a central role in cancer progression by inducing the transformation of normal cells into cancerous ones and supporting the phenotypic transformation of tumor cells. This transformation promotes tumor growth by stimulating proliferative signaling through pathways such as MAP/ERK, PI3K/AKT, and WNT. Sustained autocrine or paracrine signaling mediated by cancer EVs provides tumor cells with a proliferative advantage [100]. For example, EVs from GBM cells have been shown to enhance tumor growth by transferring oncogenic proteins to neighboring glioma cells, activating growth‐promoting pathways. Studies involving GBM cells injected into mice demonstrated that EVs containing specific proteins enhanced tumor growth compared to EVs lacking those proteins [98]. Additionally, EVs from aggressive cancer cells, such as MDA‐MB231 breast cancer cells, have been shown to transfer malignancy traits like anchorage‐independent growth and survival under nutrient‐limiting conditions to normal cells. EVs also play a critical role in resisting apoptosis. GBM EVs, for example, have been found to carry splicing factors that induce the expression of oncogenic gene isoforms in recipient cells, promoting cell survival. Additionally, microRNAs (miRNAs) in EVs, such as miR‐1246 in breast cancer EVs and miR‐205 in cholangiocarcinoma EVs, have been implicated in promoting cell proliferation and suppressing apoptosis [100].

### 3.2 Evading Immune Responses

One of the hallmark abilities of cancer cells is their capacity to evade immune detection and destruction. TDEs contribute to this immune evasion by releasing immunosuppressive EVs that interact with immune cells in the TME [98, 100]. Many of these EVs contain Fas Ligand (FasL), which induces apoptosis in activated anti‐tumor immune cells, such as T‐cells, and reduces the activity of NK cells [98].

Studies have shown that TDEs can carry immune‐modulating proteins such as PD‐L1, which binds to PD‐1 receptors on cytotoxic T‐cells, blocking their activation and proliferation. For example, GBM‐derived EVs expressing PD‐L1 can directly inhibit T‐cell function [101]. Additionally, EVs from breast cancer cells have been shown to suppress T‐cell activation by delivering TGF‐β, which modulates T‐cell behavior through the TGF‐β/Smad signaling pathway.

TDEs also influence the polarization of immune cells, such as macrophages. For example, EVs released by HCC cells have been shown to contain lncRNA TUC339, which regulates macrophage activation and induces M2 macrophage polarization. M2‐like macrophages are known to support tumor growth by promoting immune suppression and tissue remodeling [100]. Furthermore, TDEs can drive the expansion of regulatory T cells (Tregs) and the differentiation of MDSCs, both of which suppress effector T‐cell activation and contribute to immune suppression in the TME [100, 102].

### 3.3 Reprogramming Energy Metabolism

Cancer cells rely on altered energy metabolism to support rapid growth and proliferation, and EVs play a role in this metabolic reprogramming. TDEs can increase the production of metabolic acids, contributing to the acidification of the TME, which enhances the secretion and uptake of EVs [98]. For instance, EVs from adriamycin‐resistant breast cancer cells contain high levels of glutathione S‐transferase P1 (GSTP1), a metabolic enzyme that helps detoxify harmful chemicals and reprogram glucose metabolism in recipient cells [100]. Moreover, EVs from breast cancer cells can transfer miR‐122 to non‐tumor cells, promoting cancer progression by altering glucose metabolism. Another example involves miRNA‐105, which is found in tumor‐derived EVs from breast cancer cells and enhances glutamine and glucose metabolism in CAFs, providing energy to tumor cells. In nutrient‐scarce environments, CAFs expel metabolic waste products such as lactate, which are then taken up by cancer cells to fuel oxidative phosphorylation, creating a metabolic symbiosis that supports tumor growth. Cancer cells stimulate the expression of HIF1a to support this relationship, creating “pseudo‐hypoxia” conditions that then promote the release of exosomes in a calcium‐dependent manner via MCT1, supporting glycolysis and facilitating tumor growth and survival [103].

### 3.4 Angiogenesis Formation

Angiogenesis, the formation of new blood vessels, is a critical process in tumor development, allowing tumors to grow beyond a few millimeters by ensuring a supply of oxygen and nutrients. EVs play a significant role in this process by delivering angiogenic factors to endothelial cells in the TME [104]. Studies have demonstrated that hypoxic tumor cells release exosomes that contribute to angiogenesis by carrying pro‐angiogenic factors like tissue factor VIIa, which activates the ERK1/2 pathway and upregulates heparin‐binding EGF‐like growth factor [96]. Additionally, TDEs can contain Delta‐like 4 (Dll4), a molecule that promotes angiogenesis by suppressing the Notch signaling pathway, which normally inhibits the development of new blood vessels. In HCC, exosomes carrying miR‐210 have been shown to promote angiogenesis by inhibiting negative regulators such as SMAD4 and STAT6 [105].

Exosomal miRNAs also regulate cancer invasion and metastasis by altering gene expression in endothelial and immune cells, creating a premetastatic environment. For example, exosomal miR‐200 has been shown to modify gene expression patterns, enhancing the metastatic potential of poorly metastatic cancer cells [96].

## 4 Role of Exosomes in the Progression of Solid Tumors

Exosomes are small vesicles that carry various cellular components, including proteins, nucleic acids, and lipids, and play a critical role in intercellular communication under both normal and pathological conditions [106]. Exosomes facilitate the transfer of miRNAs such as miR‐21, miR‐29a, miR‐221, and miR‐222, which reflect the molecular profiles of tumors. The cargo of exosomes varies depending on the cell type and origin, and recent studies have shown differences in the lipid composition of exosomes derived from cancer cells compared to those from non‐cancer cells [10].

### 4.1 Brain Tumor

Exosomes play a pivotal role in brain tumors, especially GBM, where they promote cell proliferation and inhibit apoptosis. Proteomic analyses of GBM exosomes have revealed over 1000 proteins, including pro‐angiogenic factors like interleukin‐6 (IL‐6), IL‐8, and angiogenin, which contribute to hypoxia in brain endothelial cells and tumor malignancy [23, 107]. miR‐21 and miR‐451, found in GBM exosomes, are highly expressed in the cerebrospinal fluid (CSF) and plasma of GBM patients, making them useful biomarkers for diagnosing and monitoring tumor progression. miR‐21 inhibits caspase activity, promoting cell survival, while miR‐451 regulates proliferation and migration by suppressing the AMPK signaling pathway, which adapts to metabolic stress [106, 107, 108, 109].

In neuroblastoma (NB), exosomes carry proteins involved in key biological processes like cell differentiation and proliferation. Exosomal miR‐17‐5p, secreted by MYCN‐amplified NB cells, enhances the proliferation and migration of non‐MYCN‐amplified cells, highlighting the malignant role of exosomes in tumor progression [110, 111]. Additionally, exosomal miR‐375 has been implicated in bone marrow metastasis in NB, as it promotes the differentiation of MSCs into osteogenic cells, creating a microenvironment conducive to tumor growth [112]. Targeting miR‐375 could potentially reduce metastasis, making it a promising biomarker and therapeutic target for NB patients [112, 113].

### 4.2 Liver Tumor

In HCC, exosomal miRNAs and circular RNAs (circRNAs) play crucial roles in promoting tumor proliferation and immune evasion. miR‐21 is carried by HCC‐derived exosomes [114, 115]. It Transforms HSCs into CAFs by downregulating PTEN and activating the PDK1/AKT pathway. This leads to increased secretion of pro‐angiogenic factors such as VEGF and TGF‐β, supporting angiogenesis and tumor invasion [25, 116, 117, 118]. miR‐665, another exosomal miRNA, promotes cellular proliferation through the MAPK/ERK pathway, enhancing metastatic potential [119]. Conversely, the downregulation of miR‐100b and miR‐125 in HCC exosomes activates the IGF2/AKT/mTOR pathway, essential for maintaining the stemness of HCC cells [120].

CircRNAs within HCC‐derived exosomes also facilitate immune evasion by inducing T‐cell exhaustion and promoting regulatory T cell (Treg) expansion. For instance, cirCCAR1, found in exosomes, inhibits CD8+ T‐cell activity by upregulating PD‐1 expression, leading to reduced cytotoxicity against HCC cells [19]. Additionally, circTMEM45A in HCC exosomes sponges miR‐665, promoting IGF2 expression and cell proliferation by accelerating the G1/S cell cycle transition, contributing to tumor growth [121, 122].

Exosomes also modify the TME through EMT pathways. Circ_0004277 from HCC exosomes activates the EMT pathway by stimulating mesenchymal markers like N‐cadherin, which facilitates HCC cell migration and the progression of normal cells into malignant ones [26]. Similarly, circ_0061395 enhances tumor proliferation by sponging miR‐877‐5p, leading to increased expression of the oncogene PIK3R3 [123, 124]. Circ_002136 derived from HCC cells Huh7 and HA22T cells increased proliferation, migration, and invasiveness of HCC cells through sponging of miR‐19a‐3p and downregulating RAB1A, which are members of Rab proteins needed for the trafficking of amino acids from the Golgi apparatus and endoplasmic reticulum, along with upregulation of the mTOR signaling pathway for cellular growth and migration [125].

### 4.3 Breast Cancer

Breast cancer‐derived exosomes play a critical role in tumor progression by influencing stromal cells in the TME. Exosomal miR‐135b‐5p promotes the transition of MSCs into CAFs by inhibiting TXNIP, while miR‐146a in exosomes converts normal fibroblasts into CAFs, which enhances metastasis in MCF‐7 cells [126, 127]. Additionally, exosomal Survivin from breast cancer cells transforms fibroblasts into CAFs by upregulating superoxide dismutase 1 (SOD1), contributing to tumor growth [27, 128].

Exosomal miRNAs, including miR‐21, miR‐19b‐3p, and miR‐5100, also regulate the EMT, promoting metastasis [10, 129]. miR‐1910‐3p, encapsulated in breast cancer exosomes, enhances metastasis by activating the Wnt/β‐catenin and NF‐κB signaling pathways, while downregulating tumor suppressors like myotubularin‐related protein 3 [28].

Long non‐coding RNAs (lncRNAs) in exosomes also contribute to breast cancer progression [130, 131]. For example, lncRNA BCRT1, overexpressed in breast cancer exosomes, is associated with poor prognosis and promotes macrophage polarization to a tumor‐supportive phenotype [132]. Additionally, Lnc GS1‐600G8.5, carried by exosomes, facilitates brain metastasis by disrupting the BBB, while reduced expression of lncXIST has been linked to increased brain metastasis via EMT and c‐Met signaling pathways [133].

## 5 Exosome‐Based Therapeutics for Targeting Solid Tumors

Exosomes, due to their small size, biocompatibility, and ability to carry various molecules, have gained attention as potential tools in cancer therapy. Their ability to pass through biological barriers and be engineered for targeted delivery makes them promising candidates for drug delivery systems. Tumor‐derived exosomal RNA can serve as biomarkers for cancer screening and diagnosis, while exosome‐based therapies can initiate antitumor immune responses and improve drug delivery [134, 135].

### 5.1 Brain Cancer

The BBB poses a significant challenge for the effective treatment of brain cancers such as GBM. Exosomes have emerged as potential nanocarriers for anticancer therapies due to their ability to cross the BBB. Their stability, low immunogenicity, and high loading capacity make them ideal candidates for delivering drugs or therapeutic molecules directly to the tumor site.

One promising approach uses dendritic cell‐derived exosomes, which have demonstrated immune‐related antitumor activity in GBM mouse models [136]. Additionally, exosomes from MSCs have shown potential in delivering therapeutic miRNAs to glioma cells. For example, miR‐199a delivered via MSC‐derived exosomes inhibits glioma cell proliferation and migration by downregulating AGAP2 expression, leading to increased apoptosis in glioma cells [137].

Another promising strategy involves the use of exosomal miR‐151a, which has been linked to overcoming temozolomide (TMZ) resistance in GBM. Chemo‐resistant GBM cells have lower levels of miR‐151a in their exosomes, contributing to resistance. Restoring miR‐151a in exosomes could reverse this resistance, making it a potential therapeutic target for refractory GBM [138, 139, 140].

Exosomes also offer potential for drug delivery. Studies in zebrafish and mouse models have shown that exosomes derived from glioma and brain endothelial cells can effectively deliver chemotherapeutic agents such as paclitaxel (PTX) and doxorubicin (DXR) across the BBB, reducing tumor progression and overcoming chemoresistance [24, 141]. Additionally, exosomes containing microRNA‐29a‐3p have shown promise in suppressing angiogenesis and migration in resistant gliomas, offering a novel anti‐angiogenic therapeutic approach [8].

### 5.2 Liver Cancer

Hepatocellular carcinoma is commonly treated with surgical resection, ablation, or targeted drug therapies. While conventional therapies such as sorafenib and regorafenib are widely used, exosome‐based therapies are emerging as novel strategies for enhancing treatment outcomes [142]. Dendritic cell‐derived exosomes (DEX), for instance, have shown promise in activating T cells and inducing antitumor responses in HCC by upregulating IFN‐γ and IL‐2 while downregulating inhibitory cytokines like TGF‐β and IL‐10 [143, 144].

NK cell‐derived exosomes also show potential in inducing apoptosis in HCC cells through perforin and granzyme B pathways, leading to the activation of caspases and promoting cell death [145, 146]. Moreover, engineered exosomes carrying miR‐654‐5p have been shown to induce ferroptosis in HCC cells by inhibiting HSPB1, enhancing the efficacy of sorafenib, a drug that is often hindered by resistance due to HSPB1 overexpression [147]. Combination therapies using exosomes have demonstrated enhanced therapeutic effects. For instance, dendritic cell‐derived exosomes combined with microwave ablation resulted in reduced tumor size by decreasing Tregs and increasing CD8+ T cells, a more effective outcome than either treatment alone [148, 149]. The combination of exosomes derived from dendritic cells, along with P47 (peptide targeting the HCC cells), HMGN1 (recruiting the DC), and AFP212‐A2 (exosomal anchors), on injection, suppressed tumor growth and increased the immune responses through immune‐stimulatory CD8+ effector T‐cell recruitment and IFN‐g along with a decrease in immunosuppressive factors such as TGF‐b and IL‐10 [150]. This approach highlights the potential for exosome‐based combination therapies in improving immune responses and reducing tumor progression in HCC.

### 5.3 Breast Cancer

**FIGURE 4:**
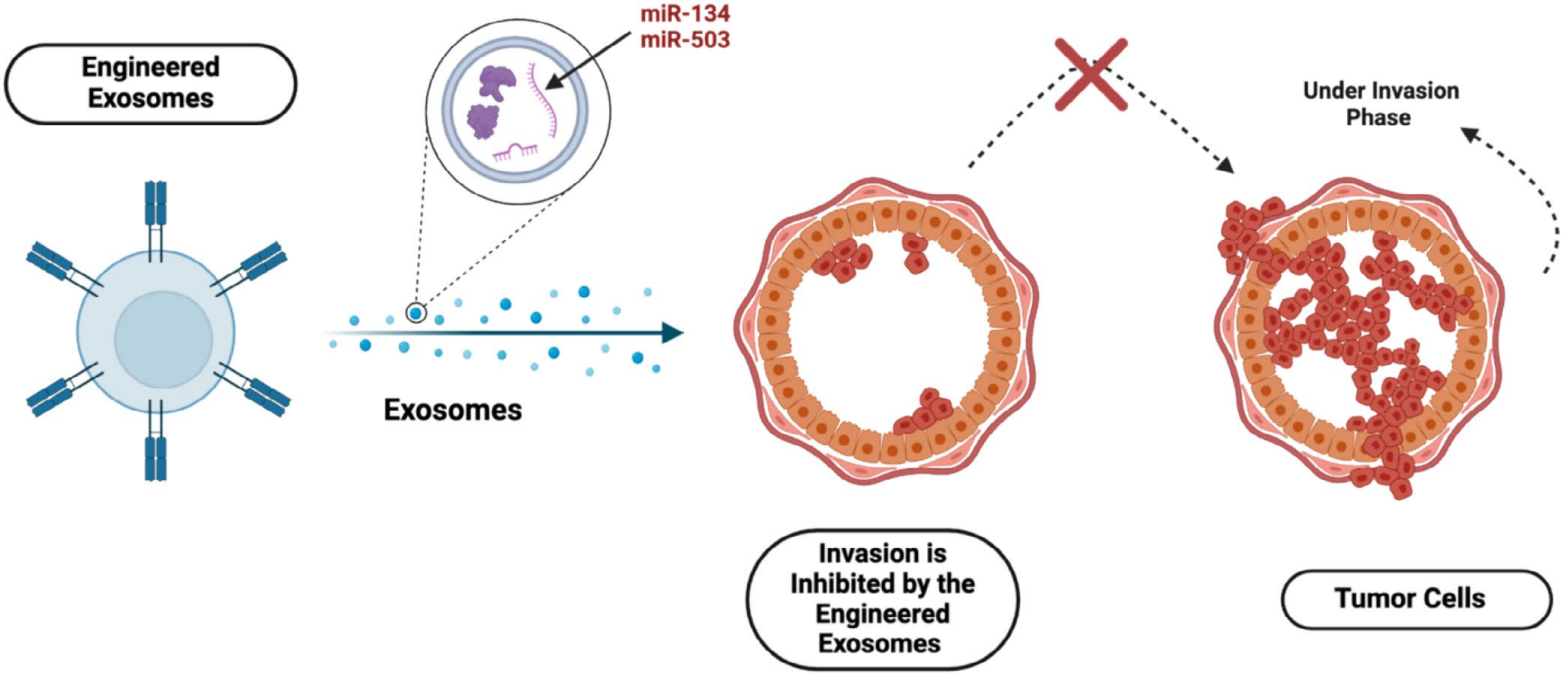
Key exosomal miRNAs (miR‐134 & miR‐503) inhibit the invasion and migration of breast cancer cells while increasing their sensitivity to anti‐HSP90 inhibitors like PU‐H71(128). Another miRNA, miR‐503, inhibits breast cancer cell invasion by targeting CCND3 and CCND2, both of which are involved in cell cycle regulation.

Breast cancer‐derived exosomes (TDEs) have garnered significant interest for their potential in diagnosis and therapy. Engineered exosomes, enriched with miRNAs, have been explored as therapeutic alternatives. For example, exosomal miR‐134 has been shown to inhibit the invasion and migration of breast cancer cells while increasing their sensitivity to anti‐HSP90 inhibitors like PU‐H71 [151]. Another miRNA, miR‐503, inhibits breast cancer cell invasion by targeting CCND3 and CCND2, both of which are involved in cell cycle regulation, as shown in Figure 4 [152, 153].

Exosomes also play a role in modulating the TME. Studies have shown that exosomes carrying miR‐135b‐5p can induce the transition of MSCs into CAFs, promoting tumor growth and metastasis [104]. Additionally, exosomal long non‐coding RNA (lncRNA) BCRT1, overexpressed in breast cancer cells, promotes macrophage polarization and enhances tumor progression [154, 155, 156]. The potential of exosomes in breast cancer treatment is evident in their ability to carry therapeutic molecules and modulate the TME. However, further research is needed to develop reliable protocols for isolating, characterizing, and utilizing exosomes in clinical applications.

## 6 Clinical Applications of Exosome Biomarkers

Traditional cancer diagnostic methods, primarily imaging and invasive biopsies, are costly, often uncomfortable, and typically detect cancer at later stages, particularly in the absence of routine screening. Late‐stage diagnosis diminishes treatment efficacy and reduces survival rates [157, 158]. This has prompted a shift towards less invasive, cost‐effective methods for early detection and continuous monitoring. Exosomes, cell‐derived EVs found in biofluids such as blood, plasma, serum, urine, and saliva, are emerging as valuable tools in this area due to their molecular contents that reflect cellular states [159, 160, 161].

Several isolation techniques for exosomes have been developed, including size‐exclusion chromatography (SEC) [162, 163, 164], ultracentrifugation, and combined approaches like ultrafiltration with SEC [165]. Recently, protocols utilizing ultracentrifugation paired with tangential flow filtration (TFF) and SEC have demonstrated higher efficiency, specificity, and reproducibility in isolating exosomes from serum [166].

Exosomes are rich in stable proteins and microRNAs, including cancer‐specific molecules that provide insight into tumor biology. Liquid biopsies analyzing these exosomal contents offer non‐invasive diagnostic and monitoring capabilities for various cancers. For instance, in brain cancers, exosomes can cross the BBB and carry glioma markers like IDH1G395A, providing a method to monitor tumor progression through blood samples [167]. Additionally, EGFRvIII, an oncogenic marker frequently upregulated in high‐grade gliomas, can be detected in serum‐derived exosomes with a sensitivity of 81.58% and specificity of 79.31% [168]. In HCC, exosomal miR‐21 is associated with tumor stage, cirrhosis, and chemotherapy resistance, making it a sensitive biomarker for HCC diagnosis compared to serum miR‐21 (Wang et al. [169], Cao et al. [170]). Breast cancer diagnostics also benefit from exosomal miRNAs; for example, miR‐939, linked to poor prognosis, is overexpressed in basal‐like and triple‐negative breast cancer subtypes [171]. Exosomal biomarkers hold promising clinical applications for non‐invasive cancer diagnosis and monitoring, supporting early detection and providing crucial insights into tumor progression and therapeutic responses.

## 7 Conclusion

Exosomes have emerged as key contributors to the progression of solid cancers by facilitating cell communication and shaping the TME. In brain tumors like GBM and NB, exosomes influence disease progression by mediating interactions within the TME. In liver cancer, the regulation of microRNAs and circRNAs via exosomes is pivotal for tumor growth and metastasis. Similarly, in breast cancer, exosomes play dual roles, contributing to both immunosuppression and metastasis. A deeper understanding of EV signaling in cancer is essential for addressing growth resistance and informing therapeutic strategies. Research has demonstrated that the cargo of EVs is highly dynamic, particularly in physiologically relevant tumor models, and evolves throughout different stages of cancer progression. However, most experimental evidence has been derived from in vitro and animal models, and there has been limited progress in clinical research. Future studies should focus on pathophysiological models in living organisms with tumors, rather than relying solely on interactions with single cells. The potential clinical implications of understanding EV dynamics are significant. A better grasp of EV signaling networks could help predict tumor progression and enable the development of innovative strategies to restore control over these networks in cancer patients. By emphasizing the clinical relevance of this research, we highlight the direct impact it can have on improving cancer treatment and patient outcomes.

## Author Contributions

**Nada Aakel:** validation (equal), writing – original draft (equal). **Rawdhah Mohammed:** writing – original draft (equal). **Assela Fathima:** writing – original draft (equal). **Rabia Kerzabi:** writing – original draft (equal). **Atiyeh Abdallah:** validation (equal), writing – review and editing (equal). **Wisam Nabeel Ibrahim:** conceptualization (lead), project administration (lead), supervision (lead), writing – review and editing (lead).

## Conflicts of Interest

The authors declare no conflicts of interest.

## Data Availability

This review article does not include any primary data. All data discussed and analyzed in this review are from previously published studies, which are cited and available through publicly accessible databases and publications. No new data were generated for this study.

## References

[1] P. S. Roy and B. J. Saikia , “Cancer and Cure: A Critical Analysis,” Indian Journal of Cancer 53, no. 3 (2016): 441–442. 28244479 10.4103/0019-509X.200658

[2] H. T. Nia , L. L. Munn , and R. K. Jain , “Physical Traits of Cancer,” Science 370, no. 6516 (2020): eaaz0868. 33122355 10.1126/science.aaz0868 PMC8274378

[3] K. E. de Visser and J. A. Joyce , “The Evolving Tumor Microenvironment: From Cancer Initiation to Metastatic Outgrowth,” Cancer Cell 41, no. 3 (2023): 374–403. 36917948 10.1016/j.ccell.2023.02.016

[4] M. A. Zaimy , N. Saffarzadeh , A. Mohammadi , et al., “New Methods in the Diagnosis of Cancer and Gene Therapy of Cancer Based on Nanoparticles,” Cancer Gene Therapy 24, no. 6 (2017): 233–243. 28574057 10.1038/cgt.2017.16

[5] K. M. Bussard , L. Mutkus , K. Stumpf , C. Gomez‐Manzano , and F. C. Marini , “Tumor‐Associated Stromal Cells as Key Contributors to the Tumor Microenvironment,” Breast Cancer Research 18, no. 1 (2016): 84. 27515302 10.1186/s13058-016-0740-2 PMC4982339

[6] D. F. Quail and J. A. Joyce , “Microenvironmental Regulation of Tumor Progression and Metastasis,” Nature Medicine 19, no. 11 (2013): 1423–1437. 10.1038/nm.3394 PMC3954707 24202395

[7] C. Li and X. Xu , “Biological Functions and Clinical Applications of Exosomal Non‐Coding RNAs in Hepatocellular Carcinoma,” Cellular and Molecular Life Sciences 76, no. 21 (2019): 4203–4219. 31300868 10.1007/s00018-019-03215-0 PMC11105530

[8] N. Zhang , F. He , T. Li , et al., “Role of Exosomes in Brain Diseases,” Frontiers in Cellular Neuroscience 15 (2021): 743353. 34588957 10.3389/fncel.2021.743353 PMC8473913

[9] Y. L. Tai , K. C. Chen , J. T. Hsieh , and T. L. Shen , “Exosomes in Cancer Development and Clinical Applications,” Cancer Science 109, no. 8 (2018): 2364–2374. 29908100 10.1111/cas.13697 PMC6113508

[10] J. Liu , L. Ren , S. Li , et al., “The Biology, Function, and Applications of Exosomes in Cancer,” Acta Pharmaceutica Sinica B 11, no. 9 (2021): 2783–2797. 34589397 10.1016/j.apsb.2021.01.001 PMC8463268

[11] T. L. Whiteside , “Tumor‐Derived Exosomes and Their Role in Cancer Progression,” Advances in Clinical Chemistry 74 (2016): 103–141. 27117662 10.1016/bs.acc.2015.12.005 PMC5382933

[12] L. M. Doyle and M. Z. Wang , “Overview of Extracellular Vesicles, Their Origin, Composition, Purpose, and Methods for Exosome Isolation and Analysis,” Cells 8, no. 7 (2019): 727. 31311206 10.3390/cells8070727 PMC6678302

[13] F. Taus , A. Meneguzzi , M. Castelli , and P. Minuz , “Platelet‐Derived Extracellular Vesicles as Target of Antiplatelet Agents. What is the Evidence?,” Frontiers in Pharmacology 10 (2019): 1256. 31780927 10.3389/fphar.2019.01256 PMC6857039

[14] H. M. ElBadre , S. E. M. El‐Deek , H. K. Ramadan , et al., “Potential Role of Human Umbilical Cord Stem Cells‐Derived Exosomes as Novel Molecular Inhibitors of Hepatocellular Carcinoma Growth,” Apoptosis 28, no. 9–10 (2023): 1346–1356. 37338718 10.1007/s10495-023-01863-z PMC10425301

[15] Q. Lin , C. R. Zhou , M. J. Bai , et al., “Exosome‐Mediated miRNA Delivery Promotes Liver Cancer EMT and Metastasis,” American Journal of Translational Research 12, no. 3 (2020): 1080–1095. 32269736 PMC7137059

[16] R. Mjelle , S. O. Dima , N. Bacalbasa , et al., “Comprehensive Transcriptomic Analyses of Tissue, Serum, and Serum Exosomes From Hepatocellular Carcinoma Patients,” BMC Cancer 19, no. 1 (2019): 1007. 31660891 10.1186/s12885-019-6249-1 PMC6816220

[17] L. Rios‐Colon , E. Arthur , S. Niture , Q. Qi , J. T. Moore , and D. Kumar , “The Role of Exosomes in the Crosstalk Between Adipocytes and Liver Cancer Cells,” Cells 9, no. 9 (2020): 1988. 32872417 10.3390/cells9091988 PMC7563540

[18] P. Famta , S. Shah , D. K. Khatri , S. K. Guru , S. B. Singh , and S. Srivastava , “Enigmatic Role of Exosomes in Breast Cancer Progression and Therapy,” Life Sciences 289 (2022): 120210. 34875250 10.1016/j.lfs.2021.120210

[19] Z. Hu , G. Chen , Y. Zhao , et al., “Exosome‐Derived circCCAR1 Promotes CD8 + T‐Cell Dysfunction and Anti‐PD1 Resistance in Hepatocellular Carcinoma,” Molecular Cancer 22, no. 1 (2023): 55. 36932387 10.1186/s12943-023-01759-1 PMC10024440

[20] M. Huang , X. Huang , and N. Huang , “Exosomal circGSE1 Promotes Immune Escape of Hepatocellular Carcinoma by Inducing the Expansion of Regulatory T Cells,” Cancer Science 113, no. 6 (2022): 1968–1983. 35396771 10.1111/cas.15365 PMC9207376

[21] Y. Zeng , S. Hu , Y. Luo , and K. He , “Exosome Cargos as Biomarkers for Diagnosis and Prognosis of Hepatocellular Carcinoma,” Pharmaceutics 15, no. 9 (2023): 2365. 37765333 10.3390/pharmaceutics15092365 PMC10537613

[22] W. Chen , Y. Quan , S. Fan , et al., “Exosome‐Transmitted Circular RNA hsa_circ_0051443 Suppresses Hepatocellular Carcinoma Progression,” Cancer Letters 475 (2020): 119–128. 32014458 10.1016/j.canlet.2020.01.022

[23] P. Skouras , A. N. Gargalionis , and C. Piperi , “Exosomes as Novel Diagnostic Biomarkers and Therapeutic Tools in Gliomas,” International Journal of Molecular Sciences 24, no. 12 (2023): 10162. 37373314 10.3390/ijms241210162 PMC10299187

[24] T. Yang , P. Martin , B. Fogarty , et al., “Exosome Delivered Anticancer Drugs Across the Blood‐Brain Barrier for Brain Cancer Therapy in *Danio rerio* ,” Pharmaceutical Research 32, no. 6 (2015): 2003–2014. 25609010 10.1007/s11095-014-1593-y PMC4520542

[25] Y. Zhou , H. Ren , B. Dai , et al., “Hepatocellular Carcinoma‐Derived Exosomal miRNA‐21 Contributes to Tumor Progression by Converting Hepatocyte Stellate Cells to Cancer‐Associated Fibroblasts,” Journal of Experimental & Clinical Cancer Research 37, no. 1 (2018): 324. 30591064 10.1186/s13046-018-0965-2 PMC6307162

[26] C. Zhu , Y. Su , L. Liu , S. Wang , Y. Liu , and J. Wu , “Circular RNA hsa_circ_0004277 Stimulates Malignant Phenotype of Hepatocellular Carcinoma and Epithelial‐Mesenchymal Transition of Peripheral Cells,” Frontiers in Cell and Developmental Biology 8 (2020): 585565. 33511111 10.3389/fcell.2020.585565 PMC7835424

[27] K. Li , T. Liu , J. Chen , H. Ni , and W. Li , “Survivin in Breast Cancer‐Derived Exosomes Activates Fibroblasts by Up‐Regulating SOD1, Whose Feedback Promotes Cancer Proliferation and Metastasis,” Journal of Biological Chemistry 295, no. 40 (2020): 13737–13752. 32709750 10.1074/jbc.RA120.013805 PMC7535909

[28] B. Wang , J. H. Mao , B. Y. Wang , et al., “Exosomal miR‐1910‐3p Promotes Proliferation, Metastasis, and Autophagy of Breast Cancer Cells by Targeting MTMR3 and Activating the NF‐κB Signaling Pathway,” Cancer Letters 489 (2020): 87–99. 32531321 10.1016/j.canlet.2020.05.038

[29] F. Zhao , L. Cheng , Q. Shao , et al., “Characterization of Serum Small Extracellular Vesicles and Their Small RNA Contents Across Humans, Rats, and Mice,” Scientific Reports 10, no. 1 (2020): 4197. 32144372 10.1038/s41598-020-61098-9 PMC7060188

[30] H. Zhang , T. Deng , R. Liu , et al., “Exosome‐Delivered EGFR Regulates Liver Microenvironment to Promote Gastric Cancer Liver Metastasis,” Nature Communications 8 (2017): 15016. 10.1038/ncomms15016 PMC5394240 28393839

[31] P. G. Woodman and C. E. Futter , “Multivesicular Bodies: Co‐Ordinated Progression to Maturity,” Current Opinion in Cell Biology 20, no. 4 (2008): 408–414. 18502633 10.1016/j.ceb.2008.04.001 PMC2577128

[32] B. D. Grant and J. G. Donaldson , “Pathways and Mechanisms of Endocytic Recycling,” Nature Reviews. Molecular Cell Biology 10, no. 9 (2009): 597–608. 19696797 10.1038/nrm2755 PMC3038567

[33] C. Théry , S. Amigorena , G. Raposo , and A. Clayton , “Isolation and Characterization of Exosomes From Cell Culture Supernatants and Biological Fluids,” Current Protocols in Cell Biology 15, no. 7 (2006) Chapter 3:Unit 3.22. 10.1002/0471143030.cb0322s30 18228490

[34] G. van Niel , S. Charrin , S. Simoes , et al., “The Tetraspanin CD63 Regulates ESCRT‐Independent and ‐Dependent Endosomal Sorting During Melanogenesis,” Developmental Cell 21, no. 4 (2011): 708–721. 21962903 10.1016/j.devcel.2011.08.019 PMC3199340

[35] M. Tschuschke , I. Kocherova , A. Bryja , et al., “Inclusion Biogenesis, Methods of Isolation and Clinical Application of Human Cellular Exosomes,” Journal of Clinical Medicine 9, no. 2 (2020): 436. 32041096 10.3390/jcm9020436 PMC7074492

[36] W. N. Ibrahim , A. A. Doolaanea , and M. S. B. Bin Abdull Rasad , “Effect of shRNA Mediated Silencing of YB‐1 Protein on the Expression of Matrix Collagenases in Malignant Melanoma Cell In Vitro,” Cells 7, no. 1 (2018): 7. 29320405 10.3390/cells7010007 PMC5789280

[37] H. Shao , H. Im , C. M. Castro , X. Breakefield , R. Weissleder , and H. Lee , “New Technologies for Analysis of Extracellular Vesicles,” Chemical Reviews 118, no. 4 (2018): 1917–1950. 29384376 10.1021/acs.chemrev.7b00534 PMC6029891

[38] J. S. Schorey , Y. Cheng , P. P. Singh , and V. L. Smith , “Exosomes and Other Extracellular Vesicles in Host‐Pathogen Interactions,” EMBO Reports 16, no. 1 (2015): 24–43. 25488940 10.15252/embr.201439363 PMC4304727

[39] R. Zhao , X. Chen , H. Song , Q. Bie , and B. Zhang , “Dual Role of MSC‐Derived Exosomes in Tumor Development,” Stem Cells International 2020 (2020): 8844730. 32963552 10.1155/2020/8844730 PMC7499322

[40] X. Zhang , X. Yuan , H. Shi , L. Wu , H. Qian , and W. Xu , “Exosomes in Cancer: Small Particle, Big Player,” Journal of Hematology & Oncology 8 (2015): 83. 26156517 10.1186/s13045-015-0181-x PMC4496882

[41] L. Moeinzadeh , I. Razeghian‐Jahromi , Z. Zarei‐Behjani , Z. Bagheri , and M. Razmkhah , “Composition, Biogenesis, and Role of Exosomes in Tumor Development,” Stem Cells International 2022 (2022): 8392509. 36117723 10.1155/2022/8392509 PMC9481374

[42] W. Olejarz , A. Dominiak , A. Żołnierzak , G. Kubiak‐Tomaszewska , and T. Lorenc , “Tumor‐Derived Exosomes in Immunosuppression and Immunotherapy,” Journal of Immunological Research 2020 (2020): 6272498. 10.1155/2020/6272498 PMC7261328 32537468

[43] Y. Yang , C. W. Li , L. C. Chan , et al., “Exosomal PD‐L1 Harbors Active Defense Function to Suppress T Cell Killing of Breast Cancer Cells and Promote Tumor Growth,” Cell Research 28, no. 8 (2018): 862–864. 29959401 10.1038/s41422-018-0060-4 PMC6082826

[44] T. L. Whiteside , “Immune Modulation of T‐Cell and NK (Natural Killer) Cell Activities by TEXs (Tumour‐Derived Exosomes),” Biochemical Society Transactions 41, no. 1 (2013): 245–251. 23356291 10.1042/BST20120265 PMC3721347

[45] M. Poggio , T. Hu , C. C. Pai , et al., “Suppression of Exosomal PD‐L1 Induces Systemic Anti‐Tumor Immunity and Memory,” Cell 177, no. 2 (2019): 414–427.e13. 30951669 10.1016/j.cell.2019.02.016 PMC6499401

[46] J. Monypenny , H. Milewicz , F. Flores‐Borja , et al., “ALIX Regulates Tumor‐Mediated Immunosuppression by Controlling EGFR Activity and PD‐L1 Presentation,” Cell Reports 24, no. 3 (2018): 630–641. 30021161 10.1016/j.celrep.2018.06.066 PMC6077252

[47] H. G. Zhang , H. Kim , C. Liu , et al., “Curcumin Reverses Breast Tumor Exosomes Mediated Immune Suppression of NK Cell Tumor Cytotoxicity,” Biochimica et Biophysica Acta 1773, no. 7 (2007): 1116–1123. 17555831 10.1016/j.bbamcr.2007.04.015 PMC2577190

[48] S. Gao , F. Mao , B. Zhang , et al., “Mouse Bone Marrow‐Derived Mesenchymal Stem Cells Induce Macrophage M2 Polarization Through the Nuclear Factor‐κB and Signal Transducer and Activator of Transcription 3 Pathways,” Experimental Biology and Medicine 239, no. 3 (2014): 366–375. 24500984 10.1177/1535370213518169

[49] S. Biswas , G. Mandal , S. Roy Chowdhury , et al., “Exosomes Produced by Mesenchymal Stem Cells Drive Differentiation of Myeloid Cells Into Immunosuppressive M2‐Polarized Macrophages in Breast Cancer,” Journal of Immunology 203, no. 12 (2019): 3447–3460. 10.4049/jimmunol.1900692 PMC6994919 31704881

[50] T. Eguchi , E. A. Taha , S. K. Calderwood , and K. Ono , “A Novel Model of Cancer Drug Resistance: Oncosomal Release of Cytotoxic and Antibody‐Based Drugs,” Biology 9, no. 3 (2020): 47. 32150875 10.3390/biology9030047 PMC7150871

[51] Z. Liu , C. L. Lu , L. P. Cui , et al., “MicroRNA‐146a Modulates TGF‐β1‐Induced Phenotypic Differentiation in Human Dermal Fibroblasts by Targeting SMAD4,” Archives of Dermatological Research 304, no. 3 (2012): 195–202. 21968601 10.1007/s00403-011-1178-0

[52] M. Cao , M. Seike , C. Soeno , et al., “MiR‐23a Regulates TGF‐β‐Induced Epithelial‐Mesenchymal Transition by Targeting E‐Cadherin in Lung Cancer Cells,” International Journal of Oncology 41, no. 3 (2012): 869–875. 22752005 10.3892/ijo.2012.1535 PMC3582905

[53] V. Luga and J. L. Wrana , “Tumor‐Stroma Interaction: Revealing Fibroblast‐Secreted Exosomes as Potent Regulators of Wnt‐Planar Cell Polarity Signaling in Cancer Metastasis,” Cancer Research 73, no. 23 (2013): 6843–6847. 24265274 10.1158/0008-5472.CAN-13-1791

[54] N. Cloonan , M. K. Brown , A. L. Steptoe , et al., “The miR‐17‐5p microRNA Is a Key Regulator of the G1/S Phase Cell Cycle Transition,” Genome Biology 9, no. 8 (2008): R127. 18700987 10.1186/gb-2008-9-8-r127 PMC2575517

[55] N. M. Anderson and M. C. Simon , “The Tumor Microenvironment,” Current Biology 30, no. 16 (2020): R921‐r5. 32810447 10.1016/j.cub.2020.06.081 PMC8194051

[56] R. Wei , S. Liu , S. Zhang , L. Min , and S. Zhu , “Cellular and Extracellular Components in Tumor Microenvironment and Their Application in Early Diagnosis of Cancers,” Analytical Cellular Pathology (Amsterdam) 2020 (2020): 6283796. 32377504 10.1155/2020/6283796 PMC7199555

[57] X. Lei , Y. Lei , J. K. Li , et al., “Immune Cells Within the Tumor Microenvironment: Biological Functions and Roles in Cancer Immunotherapy,” Cancer Letters 470 (2020): 126–133. 31730903 10.1016/j.canlet.2019.11.009

[58] M. M. Melssen , N. D. Sheybani , K. M. Leick , and C. L. Slingluff, Jr. , “Barriers to Immune Cell Infiltration in Tumors,” Journal for Immunotherapy of Cancer 11, no. 4 (2023): e006401. 37072352 10.1136/jitc-2022-006401 PMC10124321

[59] S. Guo and C. X. Deng , “Effect of Stromal Cells in Tumor Microenvironment on Metastasis Initiation,” International Journal of Biological Sciences 14, no. 14 (2018): 2083–2093. 30585271 10.7150/ijbs.25720 PMC6299363

[60] A. E. Denton , E. W. Roberts , and D. T. Fearon , “Stromal Cells in the Tumor Microenvironment,” Advances in Experimental Medicine and Biology 1060 (2018): 99–114. 30155624 10.1007/978-3-319-78127-3_6

[61] C. K. Brock , K. L. Hebert , M. Artiles , et al., “A Role for Adipocytes and Adipose Stem Cells in the Breast Tumor Microenvironment and Regenerative Medicine,” Frontiers in Physiology 12 (2021): 751239. 34912237 10.3389/fphys.2021.751239 PMC8667576

[62] D. T. Chu , T. N. T. Phuong , N. L. B. Tien , et al., “The Effects of Adipocytes on the Regulation of Breast Cancer in the Tumor Microenvironment: An Update,” Cells 8, no. 8 (2019): 8. 10.3390/cells8080857 PMC6721665 31398937

[63] M. H. Sherman , “Stellate Cells in Tissue Repair, Inflammation, and Cancer,” Annual Review of Cell and Developmental Biology 34 (2018): 333–355. 10.1146/annurev-cellbio-100617-062855 30028641

[64] A. G. Quiroz Reyes , S. A. Lozano Sepulveda , N. Martinez‐Acuña , et al., “Cancer Stem Cell and Hepatic Stellate Cells in Hepatocellular Carcinoma,” Technology in Cancer Research & Treatment 22 (2023): 15330338231163677. 36938618 10.1177/15330338231163677 PMC10028642

[65] H. Saman , S. S. Raza , S. Uddin , and K. Rasul , “Inducing Angiogenesis, a Key Step in Cancer Vascularization, and Treatment Approaches,” Cancers (Basel) 12, no. 5 (2020): 1172. 32384792 10.3390/cancers12051172 PMC7281705

[66] N. V. Popova and M. Jücker , “The Functional Role of Extracellular Matrix Proteins in Cancer,” Cancers (Basel) 14, no. 1 (2022): 238. 35008401 10.3390/cancers14010238 PMC8750014

[67] A. D. Theocharis , S. S. Skandalis , C. Gialeli , and N. K. Karamanos , “Extracellular Matrix Structure,” Advanced Drug Delivery Reviews 97 (2016): 4–27. 26562801 10.1016/j.addr.2015.11.001

[68] Y. Jin , J. Xing , K. Xu , D. Liu , and Y. Zhuo , “Exosomes in the Tumor Microenvironment: Promoting Cancer Progression,” Frontiers in Immunology 13 (2022): 1025218. 36275738 10.3389/fimmu.2022.1025218 PMC9584056

[69] H. Ungefroren , “Autocrine TGF‐β in Cancer: Review of the Literature and Caveats in Experimental Analysis,” International Journal of Molecular Sciences 22, no. 2 (2021): 977. 33478130 10.3390/ijms22020977 PMC7835898

[70] W. H. Yang , J. H. Cha , W. Xia , et al., “Juxtacrine Signaling Inhibits Antitumor Immunity by Upregulating PD‐L1 Expression,” Cancer Research 78, no. 14 (2018): 3761–3768. 29789418 10.1158/0008-5472.CAN-18-0040 PMC6050079

[71] K. Räsänen and M. Herlyn , “Paracrine Signaling Between Carcinoma Cells and Mesenchymal Stem Cells Generates Cancer Stem Cell Niche via Epithelial‐Mesenchymal Transition,” Cancer Discovery 2, no. 9 (2012): 775–777. 22969117 10.1158/2159-8290.CD-12-0312 PMC3646332

[72] S. Ghalehbandi , J. Yuzugulen , M. Z. I. Pranjol , and M. H. Pourgholami , “The Role of VEGF in Cancer‐Induced Angiogenesis and Research Progress of Drugs Targeting VEGF,” European Journal of Pharmacology 949 (2023): 175586. 36906141 10.1016/j.ejphar.2023.175586

[73] M. Monje , “Synaptic Communication in Brain Cancer,” Cancer Research 80, no. 14 (2020): 2979–2982. 32381657 10.1158/0008-5472.CAN-20-0646 PMC7367763

[74] S. Kita and I. Shimomura , “Extracellular Vesicles as an Endocrine Mechanism Connecting Distant Cells,” Molecules and Cells 45, no. 11 (2022): 771–780. 36380729 10.14348/molcells.2022.0110 PMC9676990

[75] A. Dominiak , B. Chełstowska , W. Olejarz , and G. Nowicka , “Communication in the Cancer Microenvironment as a Target for Therapeutic Interventions,” Cancers (Basel) 12, no. 5 (2020): 1232. 32422889 10.3390/cancers12051232 PMC7281160

[76] J. Maia , S. Caja , M. C. Strano Moraes , N. Couto , and B. Costa‐Silva , “Exosome‐Based Cell‐Cell Communication in the Tumor Microenvironment,” Frontiers in Cell and Development Biology 6 (2018): 18. 10.3389/fcell.2018.00018 PMC5826063 29515996

[77] B. L. Brücher and I. S. Jamall , “Cell‐Cell Communication in the Tumor Microenvironment, Carcinogenesis, and Anticancer Treatment,” Cellular Physiology and Biochemistry 34, no. 2 (2014): 213–243. 25034869 10.1159/000362978

[78] M. P. Bebelman , M. J. Smit , D. M. Pegtel , and S. R. Baglio , “Biogenesis and Function of Extracellular Vesicles in Cancer,” Pharmacology & Therapeutics 188 (2018): 1–11. 29476772 10.1016/j.pharmthera.2018.02.013

[79] R. Castillo‐Sanchez , A. Churruca‐Schuind , M. Martinez‐Ival , and E. P. Salazar , “Cancer‐Associated Fibroblasts Communicate With Breast Tumor Cells Through Extracellular Vesicles in Tumor Development,” Technology in Cancer Research & Treatment 21 (2022): 15330338221131647. 36222020 10.1177/15330338221131647 PMC9558853

[80] W. H. Chang , R. A. Cerione , and M. A. Antonyak , “Extracellular Vesicles and Their Roles in Cancer Progression,” in Methods in Molecular Biology, vol. 2174 (Springer US, 2021), 143–170. 32813249 10.1007/978-1-0716-0759-6_10 PMC8008708

[81] Q. Feng , C. Zhang , D. Lum , et al., “A Class of Extracellular Vesicles From Breast Cancer Cells Activates VEGF Receptors and Tumour Angiogenesis,” Nature Communications 8 (2017): 14450. 10.1038/ncomms14450 PMC5316898 28205552

[82] F. Xie , X. Zhou , P. Su , et al., “Breast Cancer Cell‐Derived Extracellular Vesicles Promote CD8(+) T Cell Exhaustion via TGF‐β Type II Receptor Signaling,” Nature Communications 13, no. 1 (2022): 4461. 10.1038/s41467-022-31250-2 PMC9343611 35915084

[83] M. Dragomir , B. Chen , and G. A. Calin , “Exosomal lncRNAs as New Players in Cell‐to‐Cell Communication,” Translational Cancer Research 7, no. Suppl 2 (2018): S243‐s52. 30148073 10.21037/tcr.2017.10.46 PMC6107076

[84] W. Li , X. Wang , C. Li , T. Chen , and Q. Yang , “Exosomal Non‐Coding RNAs: Emerging Roles in Bilateral Communication Between Cancer Cells and Macrophages,” Molecular Therapy 30, no. 3 (2022): 1036–1053. 34864204 10.1016/j.ymthe.2021.12.002 PMC8899606

[85] X. Dai , Y. Xie , and M. Dong , “Cancer‐Associated Fibroblasts Derived Extracellular Vesicles Promote Angiogenesis of Colorectal Adenocarcinoma Cells Through miR‐135b‐5p/FOXO1 Axis,” Cancer Biology & Therapy 23, no. 1 (2022): 76–88. 35100092 10.1080/15384047.2021.2017222 PMC8812748

[86] T. Gener Lahav , O. Adler , Y. Zait , et al., “Melanoma‐Derived Extracellular Vesicles Instigate Proinflammatory Signaling in the Metastatic Microenvironment,” International Journal of Cancer 145, no. 9 (2019): 2521–2534. 31216364 10.1002/ijc.32521

[87] N. Tominaga , N. Kosaka , M. Ono , et al., “Brain Metastatic Cancer Cells Release microRNA‐181c‐Containing Extracellular Vesicles Capable of Destructing Blood‐Brain Barrier,” Nature Communications 6 (2015): 6716. 10.1038/ncomms7716 PMC4396394 25828099

[88] H. G. Zhang , “Emerging Concepts of Tumor Exosome‐Mediated Cell‐Cell Communication,” Anticancer Research 33, no. 5 (2013): 2347–2348.

[89] B. Soltész , G. Buglyó , N. Németh , et al., “The Role of Exosomes in Cancer Progression,” International Journal of Molecular Sciences 23, no. 1 (2021): 8. 35008434 10.3390/ijms23010008 PMC8744561

[90] M. Colletti , D. Ceglie , A. Di Giannatale , and F. Nazio , “Autophagy and Exosomes Relationship in Cancer: Friends or Foes?,” Frontiers in Cell and Developmental Biology 8 (2020): 614178. 33511121 10.3389/fcell.2020.614178 PMC7835528

[91] X. Gao , Z. Zhang , T. Mashimo , et al., “Gliomas Interact With Non‐Glioma Brain Cells via Extracellular Vesicles,” Cell Reports 30, no. 8 (2020): 2489–2500.e5. 32101730 10.1016/j.celrep.2020.01.089

[92] S. Naryzhny , A. Volnitskiy , A. Kopylov , et al., “Proteome of Glioblastoma‐Derived Exosomes as a Source of Biomarkers,” Biomedicine 8, no. 7 (2020): 216. 10.3390/biomedicines8070216 PMC7399833 32708613

[93] D. M. Gonzalez and D. Medici , “Signaling Mechanisms of the Epithelial‐Mesenchymal Transition,” Science Signaling 7, no. 344 (2014): re8. 25249658 10.1126/scisignal.2005189 PMC4372086

[94] V. Mittal , “Epithelial Mesenchymal Transition in Tumor Metastasis,” Annual Review of Pathology 13 (2018): 395–412. 10.1146/annurev-pathol-020117-043854 29414248

[95] A. Dongre and R. A. Weinberg , “New Insights Into the Mechanisms of Epithelial‐Mesenchymal Transition and Implications for Cancer,” Nature Reviews. Molecular Cell Biology 20, no. 2 (2019): 69–84. 30459476 10.1038/s41580-018-0080-4

[96] K. Meehan and L. J. Vella , “The Contribution of Tumour‐Derived Exosomes to the Hallmarks of Cancer,” Critical Reviews in Clinical Laboratory Sciences 53, no. 2 (2016): 121–131. 26479834 10.3109/10408363.2015.1092496

[97] Y. Naito , Y. Yoshioka , Y. Yamamoto , and T. Ochiya , “How Cancer Cells Dictate Their Microenvironment: Present Roles of Extracellular Vesicles,” Cellular and Molecular Life Sciences 74, no. 4 (2017): 697–713. 27582126 10.1007/s00018-016-2346-3 PMC5272899

[98] M. Kanada , M. H. Bachmann , and C. H. Contag , “Signaling by Extracellular Vesicles Advances Cancer Hallmarks,” Trends Cancer 2, no. 2 (2016): 84–94. 28741553 10.1016/j.trecan.2015.12.005

[99] A. S. Carvalho , H. Baeta , B. C. Silva , et al., “Extra‐Cellular Vesicles Carry Proteome of Cancer Hallmarks,” Frontiers in Bioscience 25, no. 3 (2020): 398–436. 10.2741/4811 31585894

[100] C. P. R. Xavier , H. R. Caires , M. A. G. Barbosa , R. Bergantim , J. E. Guimarães , and M. H. Vasconcelos , “The Role of Extracellular Vesicles in the Hallmarks of Cancer and Drug Resistance,” Cells 9, no. 5 (2020): 1141. 32384712 10.3390/cells9051141 PMC7290603

[101] M. Droste , B. K. Thakur , and B. P. Eliceiri , “Tumor‐Derived Extracellular Vesicles and the Immune System‐Lessons From Immune‐Competent Mouse‐Tumor Models,” Frontiers in Immunology 11 (2020): 606859. 33391275 10.3389/fimmu.2020.606859 PMC7772428

[102] D. Ma , X. Gao , Z. Liu , X. Lu , H. Ju , and N. Zhang , “Exosome‐Transferred Long Non‐Coding RNA ASMTL‐AS1 Contributes to Malignant Phenotypes in Residual Hepatocellular Carcinoma After Insufficient Radiofrequency Ablation,” Cell Proliferation 53, no. 9 (2020): e12795. 32722884 10.1111/cpr.12795 PMC7507479

[103] X. Fu , J. Song , W. Yan , B. M. Downs , W. Wang , and J. Li , “The Biological Function of Tumor‐Derived Extracellular Vesicles on Metabolism,” Cell Communication and Signaling: CCS 21, no. 1 (2023): 150. 37349803 10.1186/s12964-023-01111-6 PMC10286389

[104] D. Todorova , S. Simoncini , R. Lacroix , F. Sabatier , and F. Dignat‐George , “Extracellular Vesicles in Angiogenesis,” Circulation Research 120, no. 10 (2017): 1658–1673. 28495996 10.1161/CIRCRESAHA.117.309681 PMC5426696

[105] X. J. Lin , J. H. Fang , X. J. Yang , et al., “Hepatocellular Carcinoma Cell‐Secreted Exosomal MicroRNA‐210 Promotes Angiogenesis in Vitro and in Vivo,” Molecular Therapy ‐ Nucleic Acids 11 (2018): 243–252. 29858059 10.1016/j.omtn.2018.02.014 PMC5992447

[106] Y. Aili , N. Maimaitiming , Y. Mahemuti , H. Qin , Y. Wang , and Z. Wang , “Liquid Biopsy in Central Nervous System Tumors: The Potential Roles of Circulating miRNA and Exosomes,” American Journal of Cancer Research 10, no. 12 (2020): 4134–4150. 33414991 PMC7783770

[107] S. Yang , Y. Sun , W. Liu , et al., “Exosomes in Glioma: Unraveling Their Roles in Progression, Diagnosis, and Therapy,” Cancers (Basel) 16, no. 4 (2024): 823. 38398214 10.3390/cancers16040823 PMC10887132

[108] R. Shi , P. Y. Wang , X. Y. Li , et al., “Exosomal Levels of miRNA‐21 From Cerebrospinal Fluids Associated With Poor Prognosis and Tumor Recurrence of Glioma Patients,” Oncotarget 6, no. 29 (2015): 26971–26981. 26284486 10.18632/oncotarget.4699 PMC4694967

[109] J. C. Akers , V. Ramakrishnan , R. Kim , et al., “MiR‐21 in the Extracellular Vesicles (EVs) of Cerebrospinal Fluid (CSF): A Platform for Glioblastoma Biomarker Development,” PLoS One 8, no. 10 (2013): e78115. 24205116 10.1371/journal.pone.0078115 PMC3804457

[110] B. H. Haug , Ø. H. Hald , P. Utnes , et al., “Exosome‐Like Extracellular Vesicles From MYCN‐Amplified Neuroblastoma Cells Contain Oncogenic miRNAs,” Anticancer Research 35, no. 5 (2015): 2521–2530. 25964525

[111] W. Chen , X. Hao , B. Yang , et al., “MYCN‐Amplified Neuroblastoma Cell‐Derived Exosomal miR‐17‐5p Promotes Proliferation and Migration of Non‐MYCN Amplified Cells,” Molecular Medicine Reports 23, no. 4 (2021): 245. 33537818 10.3892/mmr.2021.11884 PMC7893779

[112] T. Kinoshita , T. Hanazawa , N. Nohata , Y. Okamoto , and N. Seki , “The Functional Significance of microRNA‐375 in Human Squamous Cell Carcinoma: Aberrant Expression and Effects on Cancer Pathways,” Journal of Human Genetics 57, no. 9 (2012): 556–563. 22718022 10.1038/jhg.2012.75

[113] M. Colletti , L. Tomao , A. Galardi , et al., “Neuroblastoma‐Secreted Exosomes Carrying miR‐375 Promote Osteogenic Differentiation of Bone‐Marrow Mesenchymal Stromal Cells,” Journal of Extracellular Vesicles 9, no. 1 (2020): 1774144. 32922693 10.1080/20013078.2020.1774144 PMC7448845

[114] T. Han , L. Chen , K. Li , et al., “Significant CircRNAs in Liver Cancer Stem Cell Exosomes: Mediator of Malignant Propagation in Liver Cancer?,” Molecular Cancer 22, no. 1 (2023): 197. 38053070 10.1186/s12943-023-01891-y PMC10696692

[115] M. Hu , X. Li , Z. Jiang , et al., “Exosomes and Circular RNAs: Promising Partners in Hepatocellular Carcinoma From Bench to Bedside,” Discover Oncology 14, no. 1 (2023): 60. 37154831 10.1007/s12672-023-00672-9 PMC10167081

[116] M. D. A. Paskeh , M. Entezari , S. Mirzaei , et al., “Emerging Role of Exosomes in Cancer Progression and Tumor Microenvironment Remodeling,” Journal of Hematology & Oncology 15, no. 1 (2022): 83. 35765040 10.1186/s13045-022-01305-4 PMC9238168

[117] B. Yang , X. Feng , H. Liu , et al., “High‐Metastatic Cancer Cells Derived Exosomal miR92a‐3p Promotes Epithelial‐Mesenchymal Transition and Metastasis of Low‐Metastatic Cancer Cells by Regulating PTEN/Akt Pathway in Hepatocellular Carcinoma,” Oncogene 39, no. 42 (2020): 6529–6543. 32917956 10.1038/s41388-020-01450-5 PMC7561497

[118] M. Yao , S. Liang , and B. Cheng , “Role of Exosomes in Hepatocellular Carcinoma and the Regulation of Traditional Chinese Medicine,” Frontiers in Pharmacology 14 (2023): 1110922. 36733504 10.3389/fphar.2023.1110922 PMC9886889

[119] Z. Qu , J. Wu , J. Wu , et al., “Exosomal miR‐665 as a Novel Minimally Invasive Biomarker for Hepatocellular Carcinoma Diagnosis and Prognosis,” Oncotarget 8, no. 46 (2017): 80666–80678. 29113334 10.18632/oncotarget.20881 PMC5655229

[120] H. S. Seol , Y. Akiyama , S. E. Lee , S. Shimada , and S. J. Jang , “Loss of miR‐100 and miR‐125b Results in Cancer Stem Cell Properties Through IGF2 Upregulation in Hepatocellular Carcinoma,” Scientific Reports 10, no. 1 (2020): 21412. 33293585 10.1038/s41598-020-77960-9 PMC7722933

[121] W. Chao and P. A. D'Amore , “IGF2: Epigenetic Regulation and Role in Development and Disease,” Cytokine & Growth Factor Reviews 19, no. 2 (2008): 111–120. 18308616 10.1016/j.cytogfr.2008.01.005 PMC2314671

[122] P. F. Zhang , C. Gao , X. Y. Huang , et al., “Cancer Cell‐Derived Exosomal circUHRF1 Induces Natural Killer Cell Exhaustion and May Cause Resistance to Anti‐PD1 Therapy in Hepatocellular Carcinoma,” Molecular Cancer 19, no. 1 (2020): 110. 32593303 10.1186/s12943-020-01222-5 PMC7320583

[123] Y. Yu , L. Bian , R. Liu , Y. Wang , and X. Xiao , “Circular RNA hsa_circ_0061395 Accelerates Hepatocellular Carcinoma Progression via Regulation of the miR‐877‐5p/PIK3R3 Axis,” Cancer Cell International 21, no. 1 (2021): 10. 33407443 10.1186/s12935-020-01695-w PMC7788978

[124] X. Wang , F. L. Dong , Y. Q. Wang , H. L. Wei , T. Li , and J. Li , “Exosomal circTGFBR2 Promotes Hepatocellular Carcinoma Progression via Enhancing ATG5 Mediated Protective Autophagy,” Cell Death & Disease 14, no. 7 (2023): 451. 37474520 10.1038/s41419-023-05989-5 PMC10359294

[125] P. Yuan , J. Song , F. Wang , and B. Chen , “Exosome‐Transmitted circ_002136 Promotes Hepatocellular Carcinoma Progression by miR‐19a‐3p/RAB1A Pathway,” BMC Cancer 22, no. 1 (2022): 1284. 36476239 10.1186/s12885-022-10367-z PMC9730599

[126] H. Yin , S. Yu , Y. Xie , et al., “Cancer‐Associated Fibroblasts‐Derived Exosomes Upregulate microRNA‐135b‐5p to Promote Colorectal Cancer Cell Growth and Angiogenesis by Inhibiting Thioredoxin‐Interacting Protein,” Cellular Signalling 84 (2021): 110029. 33932496 10.1016/j.cellsig.2021.110029

[127] Z. Yang , N. Zhao , J. Cui , H. Wu , J. Xiong , and T. Peng , “Exosomes Derived From Cancer Stem Cells of Gemcitabine‐Resistant Pancreatic Cancer Cells Enhance Drug Resistance by Delivering miR‐210,” Cellular Oncology (Dordrecht) 43, no. 1 (2020): 123–136. 10.1007/s13402-019-00476-6 PMC12990725 31713003

[128] A. Shefer , A. Yalovaya , and S. Tamkovich , “Exosomes in Breast Cancer: Involvement in Tumor Dissemination and Prospects for Liquid Biopsy,” International Journal of Molecular Sciences 23, no. 16 (2022): 8845. 36012109 10.3390/ijms23168845 PMC9408748

[129] H. J. Wu , M. Hao , S. K. Yeo , and J. L. Guan , “FAK Signaling in Cancer‐Associated Fibroblasts Promotes Breast Cancer Cell Migration and Metastasis by Exosomal miRNAs‐Mediated Intercellular Communication,” Oncogene 39, no. 12 (2020): 2539–2549. 31988451 10.1038/s41388-020-1162-2 PMC7310603

[130] S. S. Blancas‐Zugarazo , E. Langley , and A. Hidalgo‐Miranda , “Exosomal lncRNAs as Regulators of Breast Cancer Chemoresistance and Metastasis and Their Potential Use as Biomarkers,” Frontiers in Oncology 14 (2024): 1419808. 39148900 10.3389/fonc.2024.1419808 PMC11324554

[131] W. Zhang , Q. Wang , Y. Yang , S. Zhou , P. Zhang , and T. Feng , “The Role of Exosomal lncRNAs in Cancer Biology and Clinical Management,” Experimental & Molecular Medicine 53, no. 11 (2021): 1669–1673. 34819615 10.1038/s12276-021-00699-4 PMC8639705

[132] Y. Lu , L. Chen , L. Li , and Y. Cao , “Exosomes Derived From Brain Metastatic Breast Cancer Cells Destroy the Blood‐Brain Barrier by Carrying lncRNA GS1‐600G8.5,” BioMed Research International 2020 (2020): 7461727. 32337272 10.1155/2020/7461727 PMC7165326

[133] F. Xing , Y. Liu , S. Y. Wu , et al., “Loss of XIST in Breast Cancer Activates MSN‐c‐Met and Reprograms Microglia via Exosomal miRNA to Promote Brain Metastasis,” Cancer Research 78, no. 15 (2018): 4316–4330. 30026327 10.1158/0008-5472.CAN-18-1102 PMC6072593

[134] J. Dai , Y. Su , S. Zhong , et al., “Exosomes: Key Players in Cancer and Potential Therapeutic Strategy,” Signal Transduction and Targeted Therapy 5, no. 1 (2020): 145. 32759948 10.1038/s41392-020-00261-0 PMC7406508

[135] L. Mashouri , H. Yousefi , A. R. Aref , A. M. Ahadi , F. Molaei , and S. K. Alahari , “Exosomes: Composition, Biogenesis, and Mechanisms in Cancer Metastasis and Drug Resistance,” Molecular Cancer 18, no. 1 (2019): 75. 30940145 10.1186/s12943-019-0991-5 PMC6444571

[136] D. I. Avgoulas , K. S. Tasioulis , R. M. Papi , and A. A. Pantazaki , “Therapeutic and Diagnostic Potential of Exosomes as Drug Delivery Systems in Brain Cancer,” Pharmaceutics 15, no. 5 (2023): 1439. 37242681 10.3390/pharmaceutics15051439 PMC10221347

[137] E. Ghasempour , S. Hesami , E. Movahed , S. H. Keshel , and M. Doroudian , “Mesenchymal Stem Cell‐Derived Exosomes as a New Therapeutic Strategy in the Brain Tumors,” Stem Cell Research & Therapy 13, no. 1 (2022): 527. 36536420 10.1186/s13287-022-03212-4 PMC9764546

[138] A. Zeng , Z. Wei , W. Yan , et al., “Exosomal Transfer of miR‐151a Enhances Chemosensitivity to Temozolomide in Drug‐Resistant Glioblastoma,” Cancer Letters 436 (2018): 10–21. 30102952 10.1016/j.canlet.2018.08.004

[139] C. L. Davidson , R. Vengoji , M. Jain , S. K. Batra , and N. Shonka , “Biological, Diagnostic and Therapeutic Implications of Exosomes in Glioma,” Cancer Letters 582 (2024): 216592. 38092145 10.1016/j.canlet.2023.216592 PMC10832613

[140] A. B. Ghaemmaghami , M. Mahjoubin‐Tehran , A. Movahedpour , et al., “Role of Exosomes in Malignant Glioma: microRNAs and Proteins in Pathogenesis and Diagnosis,” Cell Communication and Signaling: CCS 18, no. 1 (2020): 120. 32746854 10.1186/s12964-020-00623-9 PMC7397575

[141] J. Wang , W. Tang , M. Yang , et al., “Inflammatory Tumor Microenvironment Responsive Neutrophil Exosomes‐Based Drug Delivery System for Targeted Glioma Therapy,” Biomaterials 273 (2021): 120784. 33848731 10.1016/j.biomaterials.2021.120784

[142] H. E. Blum , “Hepatocellular Carcinoma: Therapy and Prevention,” World Journal of Gastroenterology 11, no. 47 (2005): 7391–7400. 16437707 10.3748/wjg.v11.i47.7391 PMC4725160

[143] Z. Lu , B. Zuo , R. Jing , et al., “Dendritic Cell‐Derived Exosomes Elicit Tumor Regression in Autochthonous Hepatocellular Carcinoma Mouse Models,” Journal of Hepatology 67, no. 4 (2017): 739–748. 28549917 10.1016/j.jhep.2017.05.019

[144] Q. Rao , B. Zuo , Z. Lu , et al., “Tumor‐Derived Exosomes Elicit Tumor Suppression in Murine Hepatocellular Carcinoma Models and Humans In Vitro,” Hepatology 64, no. 2 (2016): 456–472. 26990897 10.1002/hep.28549

[145] W. Li , K. Liu , Y. Chen , M. Zhu , and M. Li , “Role of Alpha‐Fetoprotein in Hepatocellular Carcinoma Drug Resistance,” Current Medicinal Chemistry 28, no. 6 (2021): 1126–1142. 32729413 10.2174/0929867327999200729151247

[146] H. Y. Kim , H. K. Min , H. W. Song , et al., “Delivery of Human Natural Killer Cell‐Derived Exosomes for Liver Cancer Therapy: An In Vivo Study in Subcutaneous and Orthotopic Animal Models,” Drug Delivery 29, no. 1 (2022): 2897–2911. 36068970 10.1080/10717544.2022.2118898 PMC9467548

[147] J. Sun , Q. Liu , Y. Jiang , Z. Cai , H. Liu , and H. Zuo , “Engineered Small Extracellular Vesicles Loaded With miR‐654‐5p Promote Ferroptosis by Targeting HSPB1 to Alleviate Sorafenib Resistance in Hepatocellular Carcinoma,” Cell Death Discovery 9, no. 1 (2023): 362. 37777559 10.1038/s41420-023-01660-2 PMC10542782

[148] X. Zhong , Y. Zhou , Y. Cao , et al., “Enhanced Antitumor Efficacy Through Microwave Ablation Combined With a Dendritic Cell‐Derived Exosome Vaccine in Hepatocellular Carcinoma,” International Journal of Hyperthermia 37, no. 1 (2020): 1210–1218. 33100037 10.1080/02656736.2020.1836406

[149] L. Rocamora‐Reverte , F. L. Melzer , R. Würzner , and B. Weinberger , “The Complex Role of Regulatory T Cells in Immunity and Aging,” Frontiers in Immunology 11 (2020): 616949. 33584708 10.3389/fimmu.2020.616949 PMC7873351

[150] B. Zuo , Y. Zhang , K. Zhao , et al., “Universal Immunotherapeutic Strategy for Hepatocellular Carcinoma With Exosome Vaccines That Engage Adaptive and Innate Immune Responses,” Journal of Hematology & Oncology 15, no. 1 (2022): 46. 35488312 10.1186/s13045-022-01266-8 PMC9052531

[151] I. Nakase and T. Takatani‐Nakase , “Exosomes: Breast Cancer‐Derived Extracellular Vesicles; Recent Key Findings and Technologies in Disease Progression, Diagnostics, and Cancer Targeting,” Drug Metabolism and Pharmacokinetics 42 (2022): 100435. 34922046 10.1016/j.dmpk.2021.100435

[152] K. O'Brien , M. C. Lowry , C. Corcoran , et al., “miR‐134 in Extracellular Vesicles Reduces Triple‐Negative Breast Cancer Aggression and Increases Drug Sensitivity,” Oncotarget 6, no. 32 (2015): 32774–32789. 26416415 10.18632/oncotarget.5192 PMC4741729

[153] N. Bovy , B. Blomme , P. Frères , et al., “Endothelial Exosomes Contribute to the Antitumor Response During Breast Cancer Neoadjuvant Chemotherapy via microRNA Transfer,” Oncotarget 6, no. 12 (2015): 10253–10266. 25860935 10.18632/oncotarget.3520 PMC4496353

[154] Z. Meng , R. Zhang , X. Wu , Z. Piao , M. Zhang , and T. Jin , “LncRNA HAGLROS Promotes Breast Cancer Evolution Through miR‐135b‐3p/COL10A1 Axis and Exosome‐Mediated Macrophage M2 Polarization,” Cell Death & Disease 15, no. 8 (2024): 633. 39198393 10.1038/s41419-024-07020-x PMC11358487

[155] S. Lakshmi , T. A. Hughes , and S. Priya , “Exosomes and Exosomal RNAs in Breast Cancer: A Status Update,” European Journal of Cancer 144 (2021): 252–268. 33373870 10.1016/j.ejca.2020.11.033

[156] S. Halvaei , S. Daryani , S. Z. Eslami , et al., “Exosomes in Cancer Liquid Biopsy: A Focus on Breast Cancer,” Molecular Therapy ‐ Nucleic Acids 10 (2018): 131–141. 29499928 10.1016/j.omtn.2017.11.014 PMC5862028

[157] L. J. Graham , M. P. Shupe , E. J. Schneble , et al., “Current Approaches and Challenges in Monitoring Treatment Responses in Breast Cancer,” Journal of Cancer 5, no. 1 (2014): 58–68. 24396498 10.7150/jca.7047 PMC3881221

[158] T. K. Egan , “Monitoring Patients Undergoing Cancer Therapy,” Laboratory Medicine 31, no. 12 (2000): 666–671.

[159] A. Makler and W. Asghar , “Exosomal Biomarkers for Cancer Diagnosis and Patient Monitoring,” Expert Review of Molecular Diagnostics 20, no. 4 (2020): 387–400. 32067543 10.1080/14737159.2020.1731308 PMC7071954

[160] X. Wang , L. Tian , J. Lu , and I. O. Ng , “Exosomes and Cancer—Diagnostic and Prognostic Biomarkers and Therapeutic Vehicle,” Oncogene 11, no. 1 (2022): 54. 10.1038/s41389-022-00431-5 PMC9477829 36109501

[161] S. Boukouris and S. Mathivanan , “Exosomes in Bodily Fluids Are a Highly Stable Resource of Disease Biomarkers,” Proteomics. Clinical Applications 9, no. 3–4 (2015): 358–367. 25684126 10.1002/prca.201400114 PMC5502131

[162] D. D. Taylor , K. S. Lyons , and C. Gerçel‐Taylor , “Shed Membrane Fragment‐Associated Markers for Endometrial and Ovarian Cancers,” Gynecologic Oncology 84, no. 3 (2002): 443–448. 11855885 10.1006/gyno.2001.6551

[163] P. Li , M. Kaslan , S. H. Lee , J. Yao , and Z. Gao , “Progress in Exosome Isolation Techniques,” Theranostics 7, no. 3 (2017): 789–804. 28255367 10.7150/thno.18133 PMC5327650

[164] S. D. Ibsen , J. Wright , J. M. Lewis , et al., “Rapid Isolation and Detection of Exosomes and Associated Biomarkers From Plasma,” ACS Nano 11, no. 7 (2017): 6641–6651. 28671449 10.1021/acsnano.7b00549

[165] R. J. Lobb , M. Becker , S. W. Wen , et al., “Optimized Exosome Isolation Protocol for Cell Culture Supernatant and Human Plasma,” Journal of Extracellular Vesicles 4 (2015): 27031. 26194179 10.3402/jev.v4.27031 PMC4507751

[166] K. S. Visan , R. J. Lobb , S. Ham , et al., “Comparative Analysis of Tangential Flow Filtration and Ultracentrifugation, Both Combined With Subsequent Size Exclusion Chromatography, for the Isolation of Small Extracellular Vesicles,” Journal of Extracellular Vesicles 11, no. 9 (2022): e12266. 36124834 10.1002/jev2.12266 PMC9486818

[167] N. García‐Romero , J. Carrión‐Navarro , S. Esteban‐Rubio , et al., “DNA Sequences Within Glioma‐Derived Extracellular Vesicles Can Cross the Intact Blood‐Brain Barrier and Be Detected in Peripheral Blood of Patients,” Oncotarget 8, no. 1 (2017): 1416–1428. 27902458 10.18632/oncotarget.13635 PMC5352065

[168] S. V. Manda , Y. Kataria , B. R. Tatireddy , et al., “Exosomes as a Biomarker Platform for Detecting Epidermal Growth Factor Receptor‐Positive High‐Grade Gliomas,” Journal of Neurosurgery 128, no. 4 (2018): 1091–1101. 28574310 10.3171/2016.11.JNS161187

[169] H. Wang , L. Hou , A. Li , Y. Duan , H. Gao , and X. Song , “Expression of Serum Exosomal microRNA‐21 in Human Hepatocellular Carcinoma,” BioMed Research International 2014 (2014): 864894, 10.1155/2014/864894. 24963487 PMC4052145

[170] L. Q. Cao , X. W. Yang , Y. B. Chen , D. W. Zhang , X. F. Jiang , and P. Xue , “Exosomal miR‐21 Regulates the TETs/PTENp1/PTEN Pathway to Promote Hepatocellular Carcinoma Growth,” Molecular Cancer 18, no. 1 (2019): 148, 10.1186/s12943-019-1075-2. 31656200 PMC6815431

[171] M. Di Modica , V. Regondi , M. Sandri , et al., “Breast Cancer‐Secreted miR‐939 Downregulates VE‐Cadherin and Destroys the Barrier Function of Endothelial Monolayers,” Cancer Letters 384 (2017): 94–100. 27693459 10.1016/j.canlet.2016.09.013

