# Correction to Role of Exosome in Solid Cancer Progression and Its Potential Therapeutics in Cancer Treatment

**DOI:** 10.1002/cam4.71029

**Published:** 2025-07-02

Aakel N, Mohammed R, Fathima A, Kerzabi R, Abdallah A, Ibrahim WN. Role of Exosome in Solid Cancer Progression and Its Potential Therapeutics in Cancer Treatment. Cancer Medicine. 2025 May;14(9):e70941. doi: 10.1002/cam4.70941. PMID: 40344389; PMCID: PMC12063069.

The first name of the third author was misspelled. The author's name should read Aseela Fathima.

We apologize for this error.

